# The *ga3ox1b* mutation reveals the crosstalk between gibberellin and other phytohormones in controlling the growth and development of female flowers in *Cucurbita pepo*

**DOI:** 10.1093/jxb/erag324

**Published:** 2026-07-02

**Authors:** Keshav Gautam, María Segura, Alicia García, Alejandro Castro-Cegrí, Francisco Palma, Dolores Garrido, Cecilia Martínez, Manuel Jamilena

**Affiliations:** Department of Biology and Geology, Agri-Food Campus of International Excellence (CeiA3) and Research Center CIAIMBITAL, University of Almería, Almería 04120, Spain; Department of Biology and Geology, Agri-Food Campus of International Excellence (CeiA3) and Research Center CIAIMBITAL, University of Almería, Almería 04120, Spain; Department of Biology and Geology, Agri-Food Campus of International Excellence (CeiA3) and Research Center CIAIMBITAL, University of Almería, Almería 04120, Spain; Department of Plant Physiology, Sciences Faculty, University of Granada, Fuentenueva s/n, Granada 18071, Spain; Department of Plant Physiology, Sciences Faculty, University of Granada, Fuentenueva s/n, Granada 18071, Spain; Department of Plant Physiology, Sciences Faculty, University of Granada, Fuentenueva s/n, Granada 18071, Spain; Department of Biology and Geology, Agri-Food Campus of International Excellence (CeiA3) and Research Center CIAIMBITAL, University of Almería, Almería 04120, Spain; Department of Biology and Geology, Agri-Food Campus of International Excellence (CeiA3) and Research Center CIAIMBITAL, University of Almería, Almería 04120, Spain; University College Dublin, Ireland

**Keywords:** Flower development, flower opening, GA3ox, gibberellin, hormone crosstalk, ovary growth

## Abstract

*Cucurbita pepo* produces female and male unisexual flowers on the same plant, and their large size makes the species a good model for studying regulation of sex-associated traits. As male flowers appear earlier during plant development than females, a system has evolved whereby female flowers grow and open faster than male flowers, allowing synchronization for proper pollination and fertilization. Our findings establish that gibberellin (GA) drives the divergent development of female and male flowers. We characterize a loss-of-function mutation in the GA biosynthetic gene *CpGA3ox1B* that causes GA deficiency in various organs. *Cpga3ox1b*, surprisingly, only impairs female flowers, rendering them small, unopened, and sterile. Male flowers develop normally, and are fertile, like male wild-type (WT) flowers. In the WT, *CpGA3ox1B* transcription is higher in female than male flowers, causing accumulation of GA and resulting in faster growth. The dwarf plant and flower phenotypes were rescued by GA_3_, but not by ethylene or jasmonic acid, indicating that the *ga3ox1b* flower never reached the appropriate size to be sensitive to these hormones, which control petal expansion and flower opening. The GA mutation affects corolla and pistil growth rather than ethylene-regulated sex determination in the floral meristem. Opposite transcriptomic shifts in *ga3ox1b* and ethylene-insensitive *etr2b* ovaries demonstrate the mutual exclusion of ethylene and GA signaling, highlighting the role of transcription factors such as MYB62 in integrating both pathways to regulate *C. pepo* development.

## Teaser

Gibberellin drives pistil and petal growth in female *Cucurbita pepo* flowers, enabling maturation and hormone-mediated corolla expansion for proper opening and fertility.

## Introduction

Gibberellins (GAs) are primary plant hormones that regulate various aspects of plant growth and development, including plant height, and flower and fruit development (Pimenta Lange and Lange, 2006; Yamaguchi, 2008; Mutasa-Gottgens and Hedden, 2009). GA is synthesized through complex biosynthetic pathways catalyzed by ent-copalyl diphosphate synthase (CPS), ent-kaurene synthase (KS), ent-kaurene oxidase (KO), ent-kaurenoic acid oxidase (KAO), GA_20_ oxidase (GA20ox), and GA_3_ oxidase (GA3ox), whereas GA_2_ oxidase (GA2ox) is required to deactivate the GA bioactive forms (Yamaguchi, 2008; Hedden, 2020). GA20ox oxidizes GA_53_ to GA_20_ and GA_12_ to GA_9_ via GA_15_ and GA_24_. Subsequently, GA3ox synthesizes GA_4_ from GA_9_, and GA_1_ and GA_7_ from GA_20_, and finally, GA2ox inactivates GA_4_ and its precursor GA_9_ by forming GA_34_ and GA_51_, respectively. The most active forms of GA are GA_1_, GA_3_, GA_4_, and GA_7_ (Hedden, 2020).

Disruption of GA biosynthesis, perception, and signaling ultimately results in plants with dwarf phenotypes, delayed flowering, and reduced fertility (Wei *et al*., 2019; Sun *et al*., 2020; Min *et al*., 2022), whereas enhanced signaling leads to taller phenotypes and early flowering in Arabidopsis (Radi *et al*., 2006). Mutations in *GA3ox* genes result in dwarf phenotypes in maize (Chen *et al*., 2014), rice (Itoh *et al*., 2001), Arabidopsis (Chiang *et al*., 1995), and *Medicago sativa* (Dalmadi *et al*., 2008). In cucurbits, several recessive mutations in GA biosynthesis/signaling genes, including cucumber *CsVBF1*, *CsCLAVATA1*, *CsGA3ox* (*CsGy7G019320*), watermelon *ClGA3ox*, and *Cucurbita maxima CmaGA3ox*, also confer a bushy phenotype (Li *et al*., 2011; Lin *et al*., 2016; Xu *et al*., 2018; Wei *et al*., 2019; Sun *et al*., 2020; Min *et al*., 2022; Anarjan *et al*., 2023).

GA plays a critical role in floral organ development and sexual reproduction, as well as in fruit and seed set and development (Griffiths *et al*., 2006; Hu *et al*., 2008; Gallego-Giraldo *et al*., 2014). GA-deficient mutants of Arabidopsis and tomato are male sterile (Koornneef and van der Veen, 1980; Nester and Zeevaart, 1988; Goto and Pharis, 1999). A null mutation in the GA receptor genes *GID1A/B/C* was found to result in a GA-insensitive plant with a significantly dwarfed phenotype and reduced fertility (Ueguchi-Tanaka *et al*., 2005; Griffiths *et al*., 2006; Iuchi *et al*., 2007). Both *GA20ox* and *GA3ox* genes were predominantly expressed at the fruit initiation stage and were up-regulated in the ovules after fertilization (Dorcey *et al*., 2009). However, GA can be transported from one organ to another for proper growth and maturation (Goto and Pharis, 1999; Hu *et al*., 2008). Thus, the movement of GA_9_ from the ovary to the sepals and petals has been reported in cucumber flowers (Pimenta Lange and Lange, 2016).

In monoecious species of the *Cucurbitaceae* family, the differential development of female and male flowers has been mainly attributed to ethylene (ET), the key hormone responsible for sex determination. This hormone regulates the mechanisms that lead to stamen abortion during the early stages of female flower development (Martínez and Jamilena, 2021). Hormones such as auxins (AUXs) and GA could also be involved in sex determination in cucurbits. However, their functions appear to be mediated by ET (Manzano *et al*., 2013; Zhang *et al*., 2014). AUX enhances femininity through the ET pathway (Trebitsh *et al*., 1987; Zhang *et al*., 2022), and GA seems to enhance maleness by down-regulating ET biosynthesis and signaling genes in the apical shoot, but also through an ET-independent pathway that up-regulates GA signaling components such as *CsGAIP* and *CsGAMYB1* in male flowers (Zhang *et al*., 2017).

The synchronization of male and female flowering is essential for proper pollination and fertilization in monoecious species, but the control of these secondary sex-associated traits remains unclear. The evolution toward the monoecious system of *C. pepo*, in which female and male flowers occur at different phenological stages of the plant, has also resulted in differential growth of female and male flowers, allowing the upper-positioned female flowers to grow faster and open even earlier than the lower-positioned male flowers (Martínez *et al*., 2013). Using single and double mutants in ET and jasmonic acid (JA) biosynthesis and signaling genes, we found that the development and opening of *C. pepo* flowers depend on JA and ET. JA promotes the opening of female and male flowers equally, but is strongly activated by ET only in female flowers, thus accelerating the opening of female flowers compared with male flowers (Cebrián *et al*., 2023; Gautam *et al*., 2024).

Herein, we present a molecular and functional characterization of *ga3ox1b*, a novel GA-deficient mutant of *C. pepo*. The mutation causes semi-dwarfism but also reduces the growth rate of female floral organs. It also lacks female floral opening and female fertility, suggesting that GA regulation of floral organ growth and development is specific to the female flower. The results show that *CpGA3ox1B* is not involved in sex determination but is activated downstream of the sex-determining hormone ET to accelerate the growth and development of floral organs in female flowers compared with those in male flowers.

## Materials and methods

### Plant material and growth conditions

The *ga3ox1b* mutant used in this study was provided by the seed company Harris Moran (now part of HM-Clause), and was identified in a ‘vegetable marrow’ genetic background. All experiments were conducted in a multi-span greenhouse at the UAL-ANECOOP Experimental Station (Retamar, Almería, Spain; 36°51′N, 2°16′W). The plants were grown directly in ‘enarenado’ (a traditional stratified sand–mulched soil system, Gázquez *et al*., 2017) for two consecutive seasons: winter (September–December) and spring (March–July). The greenhouse was passively ventilated with flap roof windows and lateral panels. A computerized drip irrigation system was used to apply a standard nutrient solution with an electrical conductivity (EC) of 2.2 dS m^–1^ and a pH of 5.8. The planting density was two plants per m^2^, with 0.5 m spacing between emitters. Average temperatures ranged from 15 °C to 25 °C in winter and from 20 °C to 32 °C in spring, under natural photoperiod conditions.

### Evaluation of vegetative and reproductive developmental traits

The impact of the *ga3ox1b* mutation on vegetative growth was evaluated at different stages of plant development in an F_2_ segregating population. The effect of *ga3ox1b* on seed germination and early plant growth was investigated following the standard procedure developed in our laboratory (Alonso *et al*., 2024).

At an early stage of development, hypocotyl length, root length, hypocotyl weight, and root weight were recorded in 110 seedlings at the 3–4 leaf stage. At the adult plant stage, leaf length, flower pedicel length, and nodes up to 15 cm were recorded in 25 plants of each genotype.

Reproductive development was assessed by recording the number of male nodes until the first female flower and the number of male or female flowers per plant in the first 30 nodes of the plant. Since the mutation affects the development of female flowers, corolla and ovary growth rates were compared in the wild type (WT) and *ga3ox1b* mutant, measuring width and length every alternate day for up to 22 d in 20 flowers of each genotype, starting from a bud size of 2 cm. Since the *ga3ox1b* mutant is female sterile, the anthesis time was standardized based on the number of days required for a 2 cm female WT bud to reach anthesis.

To examine the inheritance of the *ga3ox1b* mutation, we used the χ^2^ test to test for deviation from the expected 3:1 segregation ratio in the F_2_ and BC_1_S_1_ segregating populations.

### Identification of the causal mutation in the *ga3ox1b* mutant

Experiments were performed in two different F_2_ populations, one in a vegetable marrow genetic background and the other in the zucchini genetic background. These populations were derived by crossing the mutant with the lines HM (vegetable marrow) or MU-CU-16 [a highly inbred zucchini-type line provided by the COMAV-UPV, Spain, which served as the reference for the *de novo* assembly of the zucchini genome (Montero-Pau *et al*., 2018)]. A total of 220 F_2_ seeds were grown in trays until the seedling stage, at which point the phenotype was verified. Young leaves of 30 WT and 30 *ga3ox1b* (extreme phenotypes) were collected for DNA isolation. Later, *ga3ox1b* and WT pools were formed by combining equal amounts of individual DNA. Genomic DNA was extracted from leaves using the cetyltrimetylammonium bromide (CTAB) method (Doyle and Doyle, 1987). The quality and purity of DNA were checked by electrophoresis on a 1% agarose gel and by the Multiskan™ Skyhigh Spectrophotometer (Thermo Scientific, Wilmington, DE, USA). DNA from each bulk sample was fragmented to an average size of 350 bp using mechanical shearing. DNA nanoball sequencing (DNBseq) technology was used for genomic DNA sequencing by BGI Genomics, generating 150 bp paired-end reads and sequenced on the DNBSEQ-G400 platform. Raw reads (pre-processed with SOAPnuke v1.5.6) were aligned to the *C. pepo* reference genome v4.1. The alignment was performed using BWA (v0.7.17, Li and Durbin, 2009) with the ‘bwa mem’ algorithm (Li, 2013), and BAM files were obtained with the ‘sort’ function of Samtools (v1.13, Danecek *et al*., 2021). Picard Tools (v2.7.1) ‘MarkDuplicates’ was used to identify and remove duplicates before indexing. Subsequently, GATK (v4.2.2.0, McKenna *et al*., 2010) ‘HaplotypeCaller’ was used with parameters ‘-ERC GVCF -G StandardAnnotation -G AS_StandardAnnotation -sample-ploidy 2’ to obtain the individual gVCF files. These were combined via ‘CombineGVCFs’ and processed through the ‘GenotypeGVCFs’ algorithm with the parameter ‘-standard-min-confidence-threshold-for-calling 10’. The QTLseq R package was used to perform quantitative trait locus (QTL)-seq analysis on the resulting VCF file using the function ‘filterSNPs’ with the following parameters: reference allele frequency (AF) <0.3, total read depth between 20 and 200, and genotype quality >50 (Mansfeld and Grumet, 2018). The single nucleotide polymorphism (SNP) index was obtained using the function ‘runQTLseqAnalysis’ (Takagi *et al*., 2013) with confidence intervals of 95% and 99%. Allelic variants within the obtained putative QTLs were filtered with alternative AF<0.3 in the WT, and AF=1 in the mutant bulk; read depth ≥10; and genotype quality ≥30. Finally, the Cucurbit Genomics Database (CuGenDB) and Integrative Genomics Viewer (IGV) software were used to select exonic variants and determine the impact of putative allelic variants on gene function (http://cucurbitgenomics.org/).

### Validation of the candidate allelic variants by high-throughput genotyping of the segregating population

Validation of co-segregation of the *ga3ox1b* phenotype and the candidate mutation was performed on 170 plants from the F_2_ segregating population. SNPs and a 3 bp insertion were genotyped using PCR allele competitive extension (PACE™) technology with the CFX96 Touch Real-Time PCR Detection System (Bio-Rad®) according to the manufacturer’s guidelines. The final reaction volume (10 μl) containing 5 μl of PACE™ 2× Master Mix Standard ROX (3CR Biosciences), 4.862 μl of 20–50 ng μl^–1^ genomic DNA, and 0.139 μl of PACE™ SNP Genotyping Assays (IDT Technologies) was used for genotyping. The 1170 bp insertion in the *CpGA3ox1B* gene was genotyped by PCR using primers flanking the insertion. Primers for genotyping are listed in Supplementary Table S1.

### External treatments with gibberellic acid, ethylene, and methyl jasmonate

The apical shoots of WT and *ga3ox1b* plants at the stage of 1–2 leaves were treated with 3 mM gibberellic acid (GA_3_), and water as control, all supplemented with 0.1% Tween-20 (Segura *et al*., 2024), in an attempt to rescue the dwarf phenotype of *ga3ox1b*. After treatment, plants were assessed for height and internode length. In addition, an external application of 5 mM methyl jasmonate (MeJA) (Cebrián *et al*., 2023; Gautam *et al*., 2024) and 5 mM GA_3_ dissolved in 0.1% Tween-20 was made to female flower buds 1–2 d before anthesis to rescue the floral mutant phenotype, as preliminary trials indicated that 3 mM was insufficient to induce flower opening. The percentage of flower opening was measured between 1 d and 6 d after treatment. The control seedlings were treated with distilled water and 0.1% Tween-20. For ET treatments, female flower buds 1–2 d before anthesis of 20 WT and 20 *ga3ox1b* plants in the flowering stage were marked. The plants were introduced into a growth chamber containing 0 (Ct) and 100 ppm of ET for 2 d, following the protocol described by Segura *et al*. (2024) and then transferred to a greenhouse under standard conditions for evaluation of flower opening.

### Phytohormone extraction and quantification

For GA determination, samples were ground in cold extraction medium (methanol/formic acid 5.2 M) (8:2, v/v) with 0.5% (w/v) butylated hydroxytoluene. The homogenates were centrifuged at 4 °C and 12 000 *g* for 15 min. The supernatants were filtered through a 0.22 µm nylon filter and transferred to vials. Each sample was injected onto a column (CORTECS™ UPLC® C18 1.6 µm, 2.1×100 mm). The solvent flow rate was 0.35 ml min^–1^ and the elution gradient was prepared with eluent A (0.05% formic acid) and eluent B (acetonitrile plus 0.05% formic acid). The chromatographic gradient was applied as follows [t (min); % A]: (0; 95%), (6; 5%), (8; 5%), (8.1; 95%). Finally, GAs were quantified using a Xevo TQ-S triple quadrupole mass spectrometer with the transitions detailed in Supplementary Table S2.

The compounds indole acetic acid (IAA), indole butyric acid (IBA), *t*-zeatin, *t*-zeatin riboside, abscisic acid (ABA), MeJA, JA, and salicylic acid (SA) were quantified using a selective hormone extraction method reported by Castro-Cegrí *et al*. (2023) with SA d4 as internal standard. Briefly, 0.5 g of ground material in liquid nitrogen was extracted in 1 ml of cold extraction buffer (isopropanol/HCl 0.1 N) (10/5; v/v) and then shaken for 1 h at 4 °C. It was then centrifuged at 12 000 *g* and 4 °C for 10 min, and 0.5 ml of dichloromethane was added to each sample and shaken vigorously. The organic phase was dried under a stream of nitrogen and stored at −20 °C under an inert atmosphere. An Acquity UPLC class system was used for solvent delivery and sample introduction. Each sample was injected into a column (Acquity UPLC BEH C18 1.7 μm, 2.1 mm×50 mm). The solvent flow rate was 0.5 ml min^–1^ and the elution gradient was prepared with eluent A (0.1% formic acid) and eluent B (acetonitrile plus 0.1% formic acid). A 5 µl aliquot of each sample was injected and the gradient profile was applied as follows [t (min); % A]: (0; 90%), (6; 5%), (8; 5%), (8.1; 90). Eluates were detected using a Xevo TQ-S triple quadrupole mass spectrometer (Waters). The ion spray voltage was set at 2500 V, the source temperature at 100 °C, and the desolvation temperature at 500 °C. The hormones were detected in the multiple reaction monitoring mode of the tandem mass spectrometer with the transitions in Supplementary Table S2.

ET production was assessed in flowers of three different stages of development: early stage (ES, petal length<25 mm); medium stage (MS, petal length ∼25–40 mm); and late stage (LS, petal length ∼40–55 mm) from WT and *ga3ox1b* adult plants growth under standard greenhouse conditions. Samples were collected and incubated at room temperature for 6 h in sealed glass containers of 250 ml. ET production was analyzed in 1 ml of gas from the container in a Varian® 3900 gas chromatograph (Varian, USA), with a flame ionization detector (FID). Calibration of the instrument was performed with standard ET gas. Three biological replicates and three measurements were taken per sample. ET production was expressed as nl g^−1^ FW 10 h^−1^.

### Transcriptomic and gene expression analysis

To investigate the molecular mechanisms underlying the *ga3ox1b* mutant phenotype, RNA-seq was performed using corolla and ovary tissues collected at the pre-anthesis stage from WT and mutant plants. Flowers of both genotypes were at the same developmental stage (LS, petal length ∼40–55 mm). For each genotype and tissue, three biological replicates were analyzed, each consisting of a pool of tissues from 4–5 independent plants.

Total RNA was extracted using the Omega Biotek EZNA® Plant RNA Kit (R6827-01) following the manufacturer’s instructions. RNA quality was assessed, and only samples with RNA integrity number (RIN) >7.8 were used for sequencing. RNA-seq was performed by BGI Genomics on the DNBseq platform by BGI genomics, generating 150 bp paired-end reads and ∼6 Gb of raw data per sample. The bioinformatic pipeline involved alignment to the *C. pepo* reference genome (v4.1) using HISAT2 (v2.2.1, Zhang *et al*., 2021) with parameters ‘-max-intronlen 500000’ and ‘-rna-strandness RF’ and transcript quantification with StringTie (v2.2.1, Pertea *et al*., 2015) using ‘-e’ and ‘-B’ options. Gene expression levels were quantified using the fragments per kilobase of transcript per million mapped reads (FPKM) method. Differentially expressed genes (DEGs) were identified with the DESeq2 R package (Love *et al*., 2014) using a false discovery rate (FDR) <0.05 and |log2(fold change) |≥1.

To further analyze gene expression patterns across floral organs and developmental stages, quantitative real-time PCR (qRT-PCR) was performed using ovary, pistil, and corolla tissues collected at different stages of flower development: ES, MS, LS, and anthesis (A). Total RNA was extracted as described above and reverse-transcribed into cDNA using the RevertAid™ cDNA synthesis kit (Thermo Fisher Scientific®). qRT-PCRs were carried out in a final volume of 10 μl using 1× Top Green qPCR Super Mix (Bio-Rad®) on a CFX-96 Touch Real-Time PCR Detection System (Bio-Rad®). Relative gene expression was calculated using the 2^−ΔΔCT^ method (Livak and Schmittgen, 2001), with *CpEF1α* and *CpACT* used as internal reference genes. Primer sequences are listed in Supplementary Table S1.

### Bioinformatics and phylogenetic analysis

Homologous protein sequences obtained from The Arabidopsis Information Resource (https://www.arabidopsis.org/) and the Cucurbits Genomics Database (http://cucurbitgenomics.org/) were identified using BLAST alignment tools at NCBI (https://blast.ncbi.nlm.nih.gov/). Phylogenetic relationships between different cucurbit species and other model species were assessed using MEGA X software (Kumar *et al*., 2018) with MUSCLE alignment (Edgar, 2004) and the maximum likelihood method based on the Poisson correction model (Zuckerkandl and Pauling, 1965), with 2000 bootstrap replicates. The phylogenetic tree was represented with iTol (https://itol.embl.de/). Gene structure was visualized using the Gene Structure Display Server (GSDS) (http://gsds.gao-lab.org/Gsds_about.php).

## Results

### Identification of the *ga3ox1b* mutation

A semi-dwarf mutation, later named *ga3ox1b*, was found to affect the growth and development of plants, flowers, and fruits. Mutant plants had shorter but broader stems, compact meristems, smaller dark green leaves, and shorter leaf and flower petioles (Fig. 1A, B). Notably, no phenotypic differences were observed between the winter and spring growing seasons, confirming its stability under different environmental conditions. The corolla of the female flowers exhibited reduced growth and was unable to open, whereas the male flowers showed normal organ growth and maturation; however, they exhibited a shorter pedicel compared with the WT (Fig. 1C–E). The segregation of the mutant phenotypes was studied in the vegetable marrow and zucchini backgrounds. In both cases, the F_1_ had a WT phenotype, and the F_2_ and BC_1_ segregated 3:1 and 1:1 for the WT and mutant phenotypes, respectively (Supplementary Table S3), indicating that *ga3ox1b* is a recessive mutation.

**Fig. 1.**
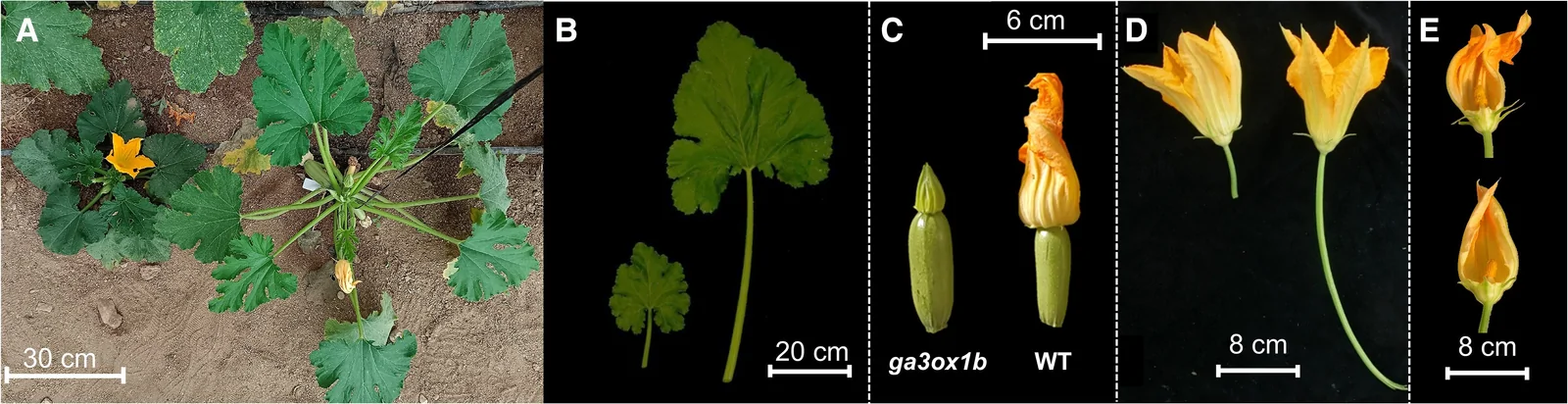
Semi-dwarf phenotype of *ga3ox1b*. (A) Visual differences between *ga3ox1b* (left) and WT 30-day-old plants. (B) Leaves from node 10 in *ga3ox1b* (left) and the WT (right). (C) Close-up of a female flower of *ga3ox1b* (flower does not open, left) and the WT (flower 1 d after opening, right). The *ga3ox1b* female flower with ovary size comparable with the WT was selected for proper comparison. (D) Male flowers of *ga3ox1b* (left) and the WT (right). Mutant male flowers open normally but have a reduced pedicel length. (E) Detail of the stamen of the male flower in *ga3ox1b* (up) and the WT (down).

To elucidate the mutation responsible for the dwarf phenotype, a segregating F_2_ population was obtained by crossing the mutant with the zucchini line MU-CU-16. This population was used to perform bulked segregant analysis (BSA) coupled with QTL-seq. A total of 30 WT and 30 *ga3ox1b* F_2_ plants were categorized into two DNA bulks (WT and *ga3ox1b*) and used for whole-genome re-sequencing. High-quality data of >90 million reads were obtained with a mapping of >99% of the reference genome, with an average depth of 48.36 and 47.59 in the WT and *ga3ox1b* bulk, respectively (Table 1). To calculate and plot the ΔSNP index against the genomic position, 1.485 and 1.481 million SNPs and indels in the WT and *ga3ox1b* bulk, respectively, were used as input to run the QTLseqr package. The study identified a putative QTL on chromosome 17 (Fig. 2A). The QTL is located between base pairs 23 698 and 8 670 776, within a peak at 5 424 917 bp on chromosome 17 that exceeds the 95% and 99% confidence thresholds, reaching a maximum G-prime value of 6.5 (Supplementary Table S4). This significant QTL is likely to be responsible for the *ga3ox1b* phenotype (Fig. 2A). The allele frequencies in the WT and *ga3ox1b* populations were used to filter out SNPs and indels. A total of 201 SNPs and indels were identified that had an alternative allele frequency of 1 in the *ga3ox1b* bulk and <0.3 in the WT bulk. Among them, 96 SNPs and indels were exonic, 21 were moderate to high effect, and 3 (indel 1 and SNPs 1 and 2) affected a conserved amino acid position in the protein. Indel 1 was an insertion of 3 bp in the gene *Cp4.1LG17g08980*, which encodes a gibberellin 3-beta-dioxygenase 1-like gene (Table 1; Fig. 2B). SNP1 was a G>A transition in the gene *Cp4.1LG17g08430* encoding the bromodomain-containing protein associated with the control of gene expression and developmental processes, while SNP2 was a T>G transversion in the gene *Cp4.1LG17g08350* encoding the protein EMSY-LIKE 4-like associated with RPP7-mediated immunity and defense (Table 1).

**Fig. 2.**
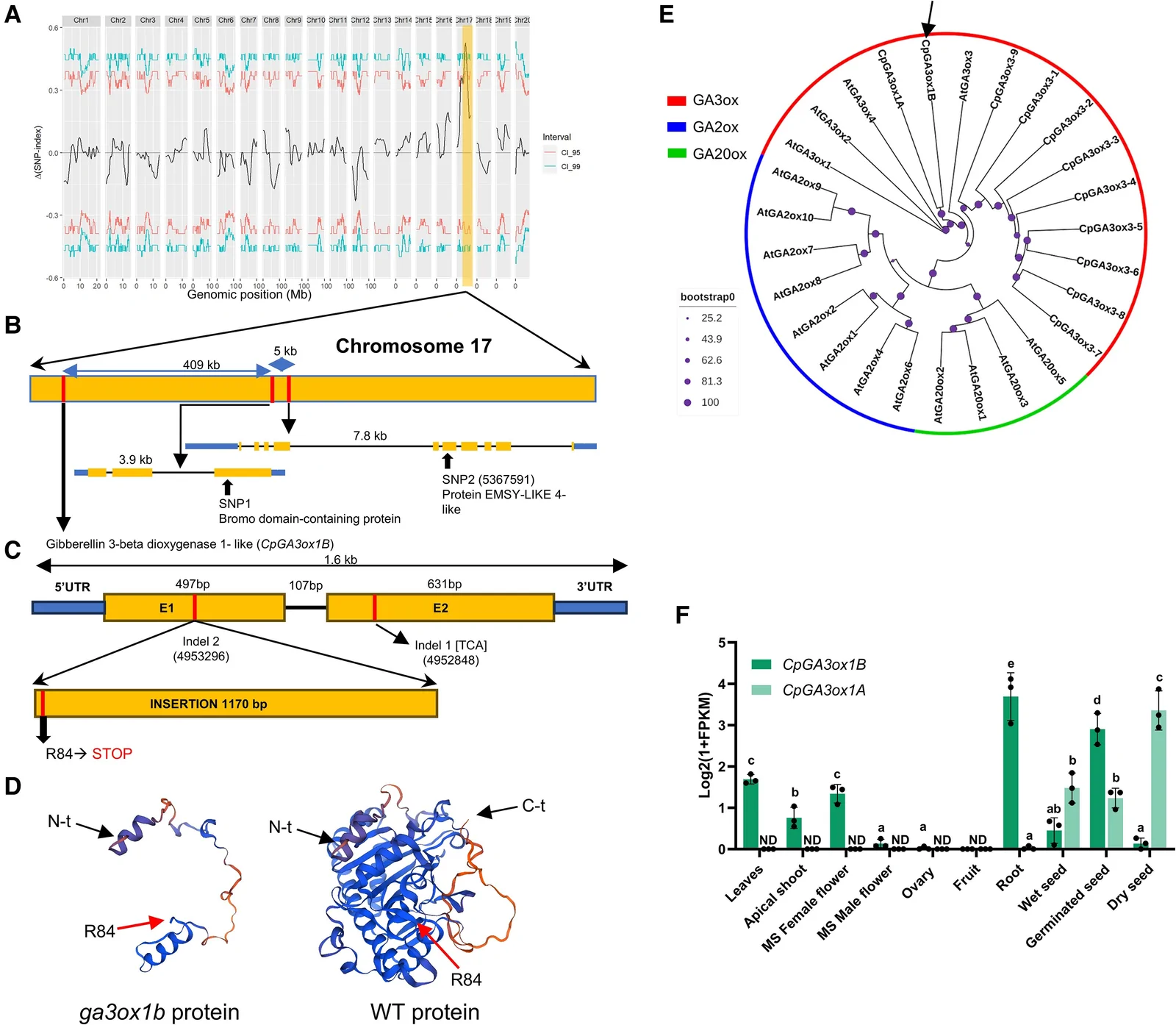
Identification of the causal mutation for the *ga3ox1b* phenotype. (A) The ΔSNP index map generated by QTL-seq analysis showed a peak on chromosome 17. (B) Representation of the chromosome 17 region, with the positions of four candidate allelic variants, SNP1, SNP2, indel 1, and indel 2. (C) Causal allelic variant detected in the gene *Cp4.1LG17g08980* (*CpGA3ox1B*), which produces an insertion of 1170 bp in the exon, resulting in the introduction of a STOP codon at residue 84. (D) Structural prediction of WT and mutant proteins. The mutant protein is missing the whole C-terminus (C-t), including the Fe2OG dioxygenase domain. (E) Phylogenetic tree of CpGA3ox proteins identified by homology with AtGA3ox, AtGA2ox, and AtGA20ox from Arabidopsis. CpGA3ox1B (arrow) and CpGA3ox1A cluster together with AtGA3ox1 from Arabidopsis. (F) Expression of *CpGA3ox1B* and its paralog *CpGA3ox1A* in different organs of WT plants. Error bars represent the SE. Different letters indicate significant differences in log2(1+FPKM) levels between different squash tissues for each gene (ANOVA, *P*≤0.05). ND, not detected.

**Table 1.** Summary of sequencing data for WT and *ga3ox1b* DNA bulks

	WT	*ga3ox1b*
**Sequencing**		
Number of reads	90 462 229	90 317 098
Mapped reads (%)	99.40	99.36
Average depth	48.36×	47.59×
Properly paired (%)	91.08	91.25
**Filtering for SNPs and indels in the QTL-17**		
Total number of SNPs and indels	1 485 429	1 481 288
Variants in chromosome 17	14 411	14 411
AF: (WT) <0.33; (*ga3ox1b*)=1	201	201
Missense mutation	96	96
Moderate to high impact	21	21
High-impact SNPs inside QTLs	0	3

Candidates for the three high-impact SNPs/indels							
	Chr	Position	Ref	Alt	Gene ID	Effect	Functional annotation
Indel 1	17	4 952 847	G	GTCA	Cp4.1LG17g08980	Asp197dup	Gibberellin 3-beta-dioxygenase 1-like
Indel 2	17	4 953 206	C	In 1170	Cp4.1LG17g08980	STOP	Gibberellin 3-beta-dioxygenase 1-like
SNP 1	17	5 362 514	G	A	Cp4.1LG17g08430	Val408Ile	Bromo domain-containing protein
SNP 2	17	5 367 591	T	G	Cp4.1LG17g08350	Asn226Thr	EMSY-LIKE 4-like protein

To confirm whether any of these three variants was the causal mutation of the phenotype, 170 plants from the F_2_ population were genotyped. Indel 1 co-segregated 100% with the *ga3ox1b* locus, while SNP1 and SNP2 showed 14% and 10% recombination with the mutant phenotype, respectively (Supplementary Table S5). Detailed analysis of the *Cp4.1LG17g08980* gene sequence in WT and *ga3ox1b* plants revealed a new insertion of 1170 bp in exon 1 (indel 2) of the mutant gene and transcript (Fig. 2C). The insertion was also found in several locations in the *C. pepo* genome, but with different sizes, suggesting that it is probably a repetitive sequence. However, it does not appear to have a coding or degenerate coding sequence from known transposons or retrotransposons. The 1170 bp insertion produced a premature stop codon and a truncated protein that most probably lost function (Fig. 2D). After genotyping indel 2 in the 170 F_2_ plants (Supplementary Table S5), the insertion was found to be 100% co-segregated with the *ga3ox1b* phenotype and was therefore likely to be the causal mutation for the mutant phenotype. The gene *Cp4.1LG17g08980*, encoding a gibberellin 3-beta-dioxygenase 1-like, clusters within the GA3ox functional clade (Fig. 2E). Within this group, the gene was identified as the best BLASTp hit for the *Arabidopsis thaliana AtGA3ox1* gene (Supplementary Table S6) and was therefore named *CpGA3ox1b*. However, this does not mean that squash protein is an AtGA3ox1 orthologue, as it also shares a high degree of homology with AtGA3ox2 and AtGA3ox4.

Because the *C. pepo* genome has undergone duplication (Montero-Pau *et al*., 2018), the identified gene *CpGA3ox1B* on chromosome 17 (designated as *Cp4.1LG17g08980*) was found to belong to the B subgenome. This gene has a paralog, *CpGA3ox1A* (*Cp4.1LG12g07890*), located in a syntenic block on chromosome 12 that belongs to subgenome A, with 67.23% similarity (https://blast.ncbi.nlm.nih.gov/Blast.cgi). The *CpGA3ox1B* and *CpGA3ox1A* paralogs have a similar molecular structure to those in other *Cucurbita* species, consisting of two exons and one intron (Supplementary Fig. S1A). A phylogenetic tree was constructed using GAox proteins identified in Arabidopsis, *C. pepo*, *Solanum lycopersicum*, *Zea mays*, and *Oryza sativa*. The CpGA3ox1B and CpGA3ox1A proteins cluster together with GA3ox in other plants (Supplementary Fig. S1B; Fig. 2E).

Previous RNA-seq data from different plant organs of the MU-CU-16 line (BioProject PRJNA1042934) showed that the *CpGA3ox1B* gene was expressed in all organs tested, including seeds, roots, leaves, apical shoots, and flowers at the medium stage of development, while *CpGA3ox1A* was only expressed in seeds (Fig. 2F). The expression level of *CpGA3ox1B* was 15 times higher in female flowers than in male flowers (Fig. 2F), suggesting that it plays an important role in the development of female flowers.

### *Ga3ox1b* interfered with vegetative plant development

To evaluate the role of *CpGA3ox1B* in early vegetative development, seed germination and seedling growth were analyzed in water under dark conditions in WT and *ga3ox1b* plants. No differences were observed between the WT and *ga3ox1b* in germination percentage after 72 h in water (Fig. 3A; Supplementary Fig. S2A). At this early stage, the *ga3ox1b* mutant exhibited a reduction in both hypocotyl length and fresh weight. However, the mutant only showed a slight decrease in root fresh weight, with no change in root length, compared with the WT (Supplementary Fig. S2B, C). During subsequent seedling growth, the mutant exhibited significantly reduced hypocotyl and shoot elongation, although the primary root length remained similar to that of the WT (Fig. 3B; Supplementary Fig. S2B, C). At 12 d after sowing, vegetative growth in *ga3ox1b* was significantly reduced compared with the WT. The most striking phenotypic difference was in shoot length, which is significantly reduced in the mutant (60%). The mutant fresh shoot and root weights were significantly reduced by 41% and 26%, respectively, compared with the WT, suggesting a higher number or wider distribution of roots, as the root length was similar to that of the WT (Fig. 3C, D). These results suggest that CpGA3ox1B positively regulates shoot elongation, as well as root and shoot fresh weight, in zucchini.

**Fig. 3.**
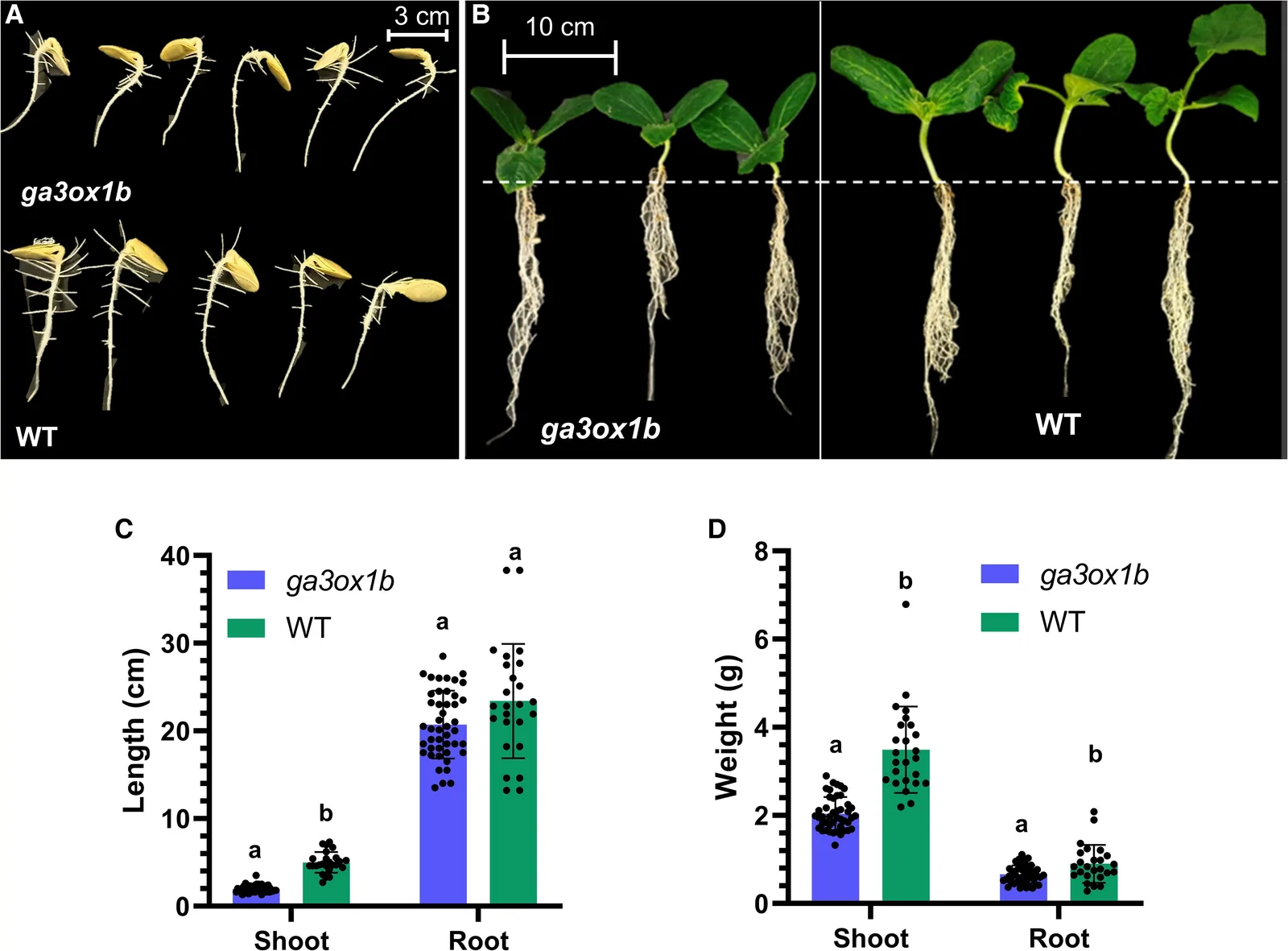
Comparative growth analysis of *ga3ox1b* and WT seedlings. (A) Seed germination after 72 h in water. (B) Difference in shoot and root size in 12-day-old seedlings of *ga3ox1b* and the WT. (C) Comparison of root and shoot length of 12-day-old plants. (D) Comparison of root and shoot fresh weight of 12-day-old plants. Error bars represent the SE. Different letters indicate significant differences between genotypes (ANOVA, *P*≤0.05).

The growth parameters of 100-day-old plants were also affected by the mutation (Table 2). Significantly reduced size was observed in the *ga3ox1b* plants in comparison with WT plants for the 20th leaf length and the 20th leaf pedicel length (Table 2). Moreover, the average length of internodes in 15 cm shoots was shorter in *ga3ox1b* than in the WT (Table 2), indicating that the dwarfism in *ga3ox1b* is due to shorter internodes rather than a reduced number of nodes, which is typical of an altered GA response.

**Table 2.** Phenotypic effect of *ga3ox1b* mutation on plant vegetative growth of 100-day-old plants

Genotype	20th leaf length (cm)	20th leaf pedicel length (cm)	Mean internode length (cm)
*ga3ox1b*	211.89±28.98^a^	228.22±26.83^a^	0.85±0.03^b^
WT	360.08±22.45^b^	315.29±20.78^b^	1.13±0.04^a^

Different letters indicate significant differences between genotypes (ANOVA, *P*≤ 0.05)

### *Ga3ox1b* interfered with female flower development, female flower opening, and female fertility

Since *ga3ox1b* affected the development and opening of female flowers, we examined the growth rate of floral organs in female flowers from 2 cm floral buds to post-anthesis flowers (Fig. 4A). When the size of WT and *ga3ox1* flowers was standardized according to the size of the ovary, the corolla and pistil of the mutant female flower were much shorter than those of the WT, but the growth of the mutant ovary was similar to or even higher than that of the WT (Fig. 4A). Although the mutation shortened the large petiole characteristic of male flowers, it did not affect the growth of other male floral organs (Fig. 1E).

**Fig. 4.**
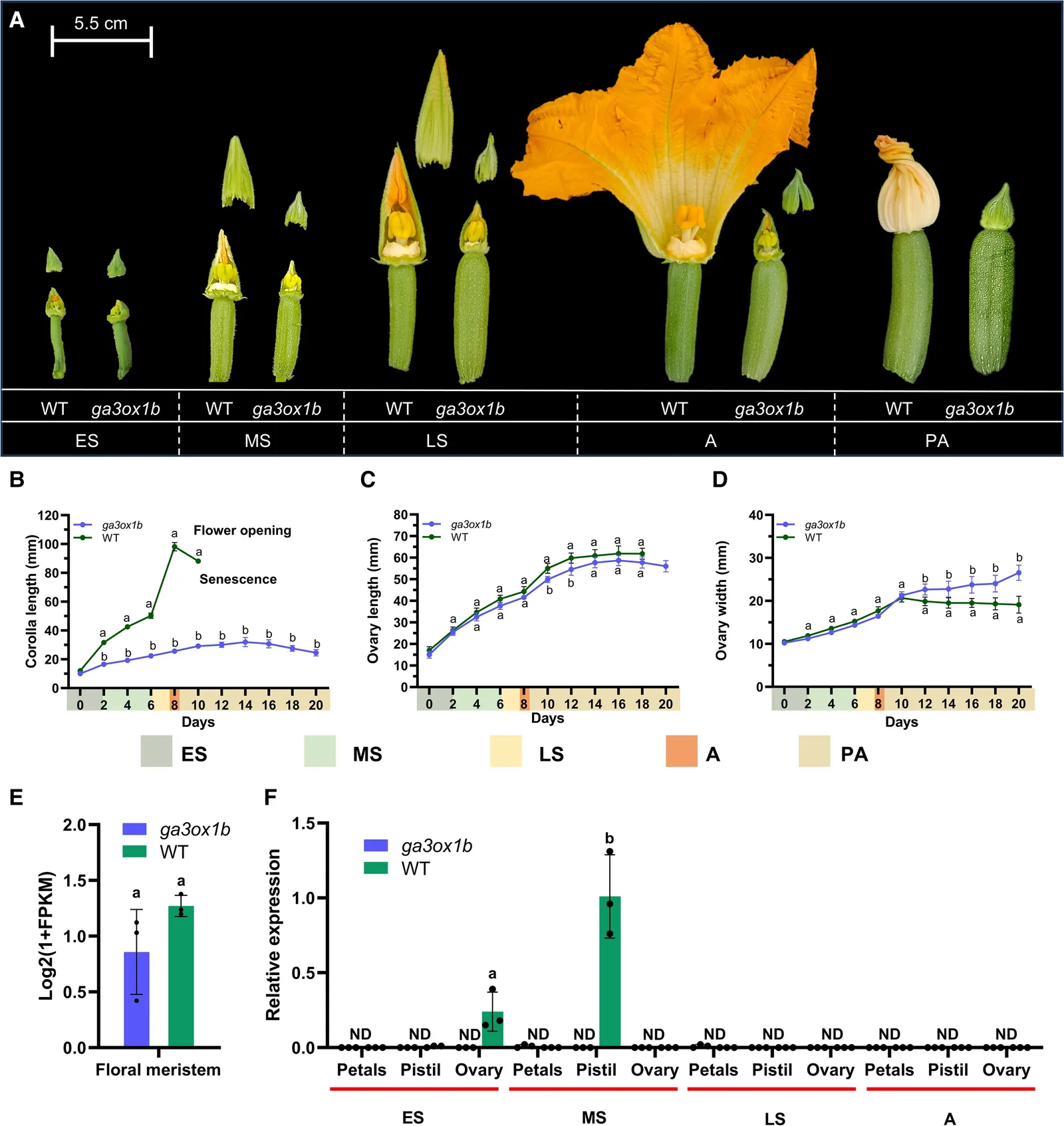
Comparison of corolla and ovary length between the WT and *ga3ox1b*. (A) Female flowers at different developmental stages: ES, early stage; MS, medium stage; LS, late stage; A, anthesis; PA, post-anthesis. (B) Corolla elongation of female flowers in the WT and *ga3ox1b*. (C) Growth rate of ovary length in the WT and *ga3ox1b*. (D) Growth rate of ovary width in the WT and *ga3ox1b*. Flowers were labeled when the bud size was 2 cm long and then measured on alternate days. Flower opening time (in orange) indicates the day when >80% of the WT flowers had opened. (E) Expression of *CpGA3ox1B* in floral meristems of the WT and *ga3ox1b.* Different letters indicate significant differences in log2 (1+FPKM) levels between genotypes (ANOVA, *P*≤0.05). (F) Relative gene expression of *CpGA3ox1B* obtained by qRT-PCR in WT and *ga3ox1b* flowers at different developmental stage. Error bars represent the SE. Different letters indicate significant differences in relative gene expression between samples and genotypes (ANOVA, *P*≤0.05). ND, not detected.

The flower opening time in *C. pepo* can be divided into three developmental stages based on the petal elongation rates: ES, MS, and LS (Cebrián *et al*., 2023; Fig. 4B, C). The growth rate of *ga3ox1b* female corolla decreased compared with that of the WT from earlier stages of development during pre-anthesis (Fig. 4B). Petal elongation in the WT was strongly induced 2 d before flower opening at the LS (Fig. 4B). However, in *ga3ox1b*, the corolla elongation rate remained lower and constant from day 2 to day 14 (Fig. 4B). The longitudinal growth rate of the WT and *ga3ox1b* ovaries was similar (Fig. 4C). The width of the ovary showed a different growth pattern from the length, growing similarly to the WT up to 2 d after anthesis, the point at which the WT ovary aborted but the *ga3ox1b* ovary continued to grow (Fig. 4D). This made the mutant fruits wider than those of the WT. At the developmental stage when the WT female flower was open and the WT and *ga3ox1b* ovaries were of the same size, the pistil of the *ga3ox1b* flower was very immature, indicating that the growth of the carpel was also inhibited in the mutant flowers compared with the WT from earlier stages of development (Fig. 4A).

The spatio-temporal expression of *CpGA3ox1B* in the floral meristems of apical shoots, and in petal, pistil, and ovary tissues of female flowers at different stages of development was determined by either RNA-seq or qRT-PCR. No significant differences in *CpGA3ox1B* expression were detected between WT and mutant apical shoots in RNA-seq data (Fig. 4E), suggesting that the gene is similarly expressed in WT and mutant floral meristems. At later stages of female flower development, the gene was mainly detected in the ovary of flowers at ES and in the pistil at MS (Fig. 4F). However, in the *ga3ox1b* mutant, transcript levels were nearly undetectable by qRT-PCR in all floral organs (Fig. 4F). This reduction in gene expression is likely to be due to nonsense-mediated mRNA decay (NMD) triggered by the premature STOP codon.

This impaired development and maturation of the mutant carpel suggests a possible female sterility of *ga3ox1b*. Self- and cross-pollinations were attempted on the *ga3ox1b* female flower buds at the presumed anthesis stage, as determined by the ovary size of the WT. At this and later stages of development, fertilization of the *ga3ox1b* flower was always unsuccessful, indicating that the mutation resulted in female sterility (Supplementary Table S7). However, the male *ga3ox1b* flower was normal and fully fertile. Stamens and pollen reached full development and maturity at anthesis and were able to produce the same number of seeds when used to pollinate WT female flowers (Supplementary Table S7).

Although *ga3ox1b* altered the growth of female floral organs, the mutation did not appear to affect the sexual expression of the plant. Indeed, the mutation did not alter the arrest of stamen primordia in female flowers (Fig. 4A), the arrest of carpel primordia in male flowers, the occurrence of the first female flower, the so-called female flowering transition, or the percentage of female or male flowers per plant (Supplementary Table S8).

### Exogenous gibberellic acid treatment partially rescued the *ga3ox1b* phenotype

In an attempt to rescue the semi-dwarf phenotype of *ga3ox1b*, the apical shoot of mutant plants with 1–2 true leaves was exogenously treated with 3 mM GA_3_ to assess their growth response. The *ga3ox1b* plants responded positively to treatment, showing an increase in internodal length, petiole length, peduncle growth, and petal size (Fig. 5A). The effect of the treatment was transient and lasted only about a week before the plants reverted to their original phenotype (Fig. 5B).

**Fig. 5.**
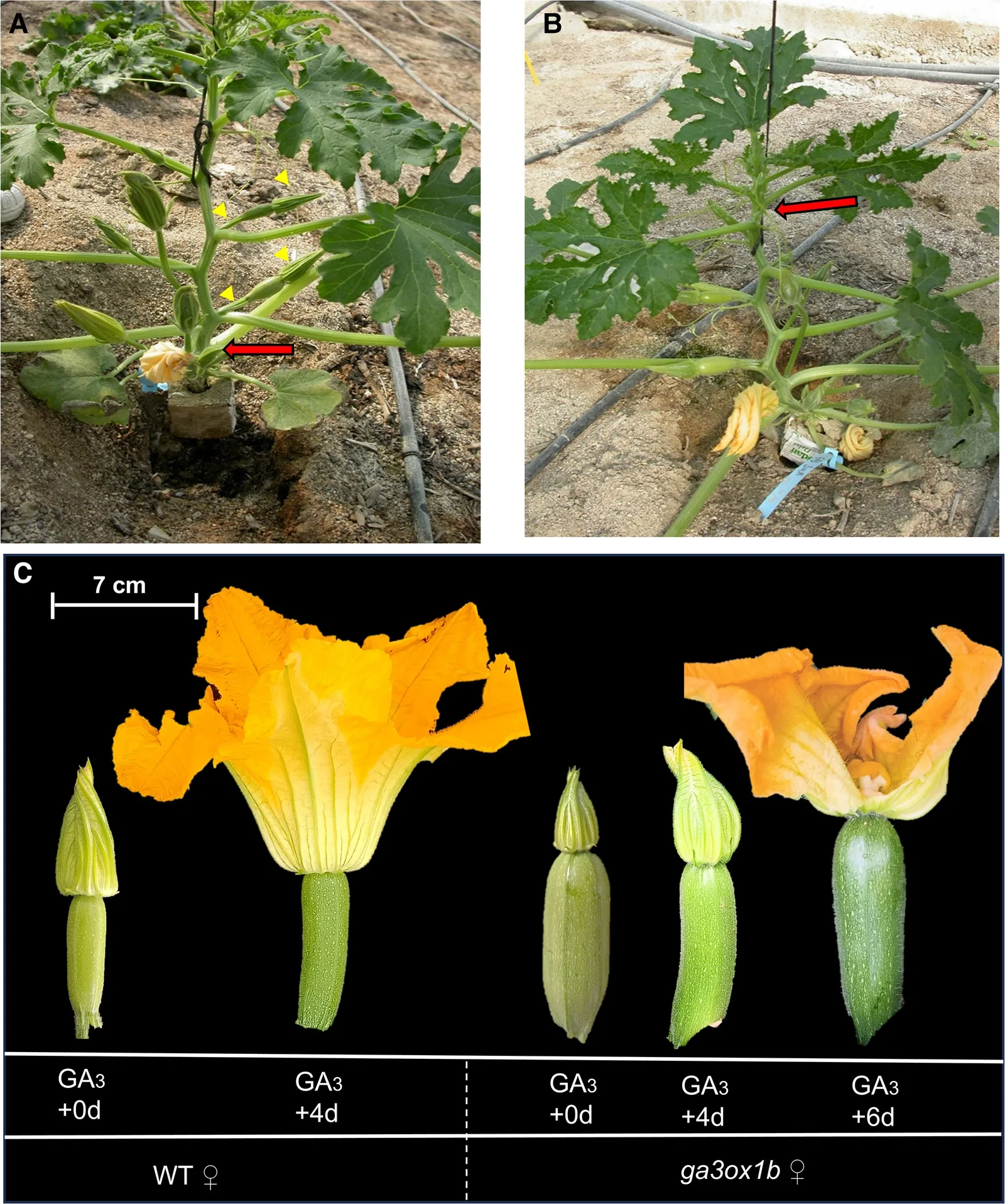
GA_3_ application rescues the flower opening defect of *ga3ox1b.* (A) Effect of 3 mM GA_3_ on *ga3ox1b* plant growth. Shown is the effect on internodal elongation and female flower peduncle and petal length (arrowheads). The application was made when plants were at the 2–3 leaf stage (arrow). (B) Loss of external GA_3_ effects. The arrow indicates loss of effect in the apical shoot. (C) Effect of 5 mM GA_3_ on the maturation and opening of the female corolla of the WT and *ga3ox1b*. Flowers were treated at a size corresponding to 1–2 d before anthesis in the WT and a similar ovary size in *ga3ox1b*. Flowers are shown on the day of treatment (left) and 4 d and 6 d after treatment (right). WT flowers at anthesis (left) are shown for comparison.

External GA_3_ was also applied to WT and mutant female flowers in the late stage of development prior to anthesis, and its effects on flower opening were assessed at 4 d and 6 d after treatment. GA_3_ application was able to induce the opening of both WT and *ga3ox1b* female flowers (Fig. 5C; Table 3). One hundred percent of the WT female flowers developed and opened 4 d after treatment. The *ga3ox1b* female flowers also responded to the treatment and were able to complete their maturation and opening 6 d after GA_3_ application (Fig. 5C; Table 3). Together, these data confirmed that *ga3ox1b* flower-defective development is due to a deficiency in GA content.

**Table 3.** Effect of gibberellin GA_3_, methyl jasmonate (MeJA), and ethylene (ET) on female flower development and opening in the WT and *ga3ox1b*

Treatment	Phenotype	No. of treated femaleflowers*^a^*	No. of flowers open at 4 DAT	No. of flowers open at 6 DAT
5 mM GA_3_	*ga3ox1b*	8	2	7
	WT	8	8	8
5 mM MeJA	*ga3ox1b*	14	0	0
	WT	9	9	9
ET	*ga3ox1b*	12	0	0
	WT	11	11	11
Control (water)	*ga3ox1b*	16	0	0
	WT	14	14	14

^*a*^Flowers were treated at pre-anthesis late stage (50–55 mm in the WT and 30 mm in *ga3ox1b*). DAT, days after treatment.

Since both JA and ET are known to regulate flower development and opening in *C. pepo* (García *et al*., 2020; Cebrián *et al*., 2021, 2023; Gautam *et al*., 2024), the sensitivity to these phytohormones was assessed by applying MeJA and gaseous ET to WT and *ga3ox1b* female flowers at the late pre-anthesis stage. The *ga3ox1b* flowers did not respond to MeJA or ET (Table 3), which suggests that they are not sensitive to ET or JA during the early stages of development when the mutant flowers were affected. It is also possible that ET and JA act upstream of GA in female flower growth and opening.

### Hormonal interplay in *ga3ox1b* developmental defects

The levels of GA, IAA, IBA, *t*-zeatin, *t*-zeatin riboside, MeJA, JA, ABA, and SA in different organs of *ga3ox1b* and the WT are shown in Fig. 6. Data confirmed the deficiency of bioactive GA in the *ga3ox1b* mutant. The levels of GA_1_, GA_3_, GA_4_, and GA_7_ were analyzed in roots, leaves, apical shoots, and female corollas and ovaries (Fig. 6A, B). GA_7_ was significantly reduced in leaves, petals, and ovaries in *ga3ox1b* plants (Fig. 6A). GA_1_ was detected only in the WT corolla and ovary, but at lower levels than GA_7_, and was undetectable in *ga3ox1b* tissues (Fig. 6B). GA_3_ and GA_4_ hormones were not detectable in any of the tissues examined.

**Fig. 6.**
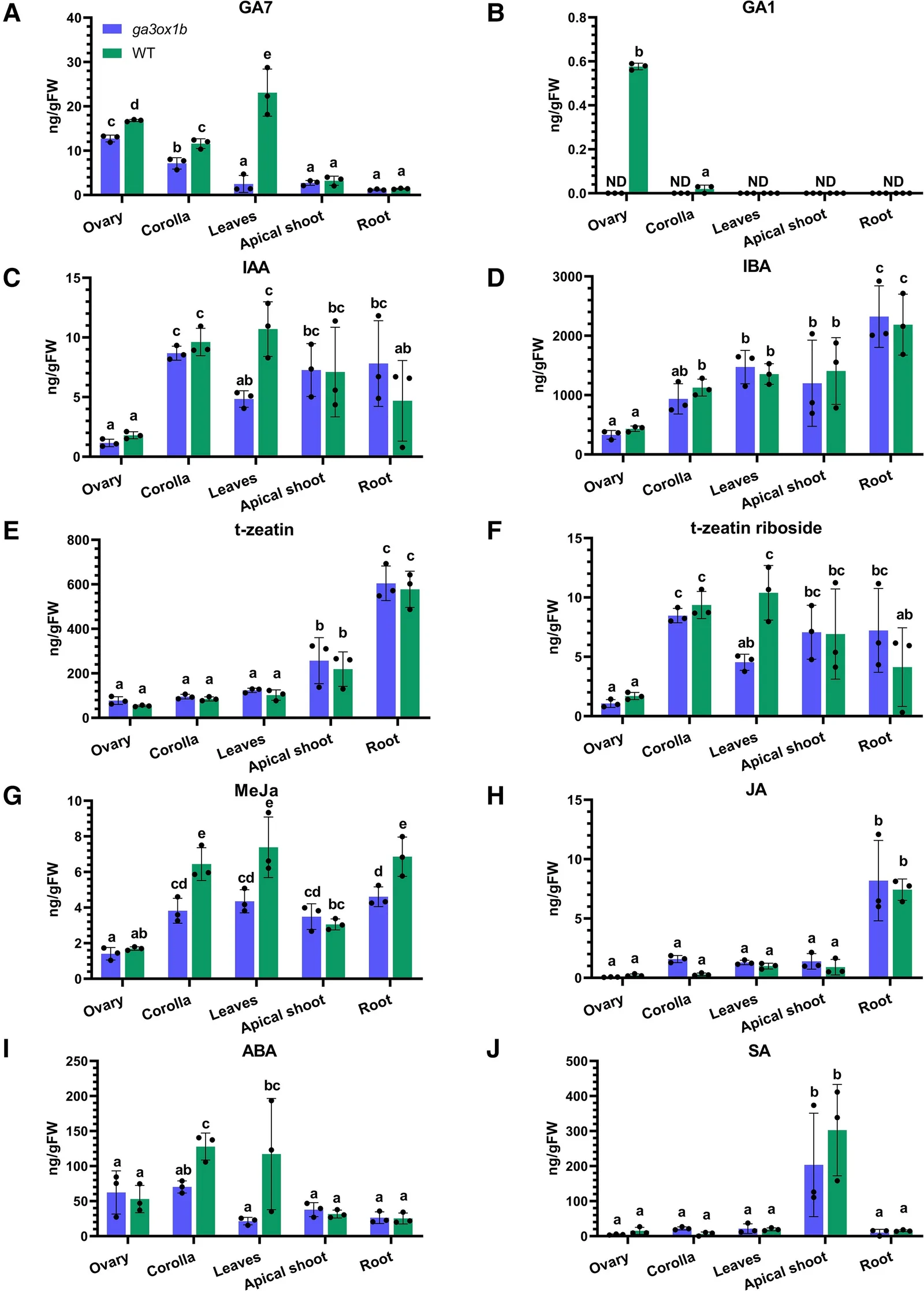
Quantification of relevant phytohormones in different plant organs of WT and *ga3ox1b* plants. (A) GA_7_, (B) GA_1_, (C) IAA, (D), IBA, (E) *t*-zeatin, (F) *t*-zeatin riboside, (G) MeJA, (H) JA, (I) ABA, and (J) SA. Assays were performed in three independent replicates. Error bars represent the SE. Different letters indicate significant differences in hormone levels between WT and *ga3ox1b* samples (ANOVA, *P*≤0.05).

Minor differences in the levels of other phytohormones were observed between *ga3ox1b* and the WT in a few organs. In leaves, the mutant had lower levels of IAA, *t*-zeatin riboside, MeJA, and ABA than WT leaves (Fig. 6). In the roots, the GA-deficient mutant *ga3ox1b* showed reduced MeJA content compared with the WT (Fig. 6G), a phytohormone reported to be an inhibitor of root elongation in *C. pepo* (Gautam *et al*., 2024). In the corolla of female flowers, the *ga3ox1b* mutation also reduced the levels of MeJA and ABA, suggesting a possible interplay between GA and these hormones in the control of petal development (Fig. 6G, I). In the ovary, the *ga3ox1b* mutation had no effect on phytohormone levels other than GA (Fig. 6).

As ET also plays a pivotal role in flower opening (García *et al*., 2020; Cebrián *et al*., 2021), ET production was measured in WT and *ga3ox1b* female and male flowers at different developmental stages (ES–LS). As expected, female flowers produced more ET than male flowers, but no difference was detected between the two genotypes, suggesting that, under our experimental conditions, the *ga3ox1b* mutation does not significantly alter the ET production required for flower opening (Supplementary Fig. S3).

### Phytohormone biosynthesis and signaling genes regulated by *ga3ox1b* in female flowers

The role of GA in vegetative organ elongation is well known in several species. However, its participation in the differential development of floral organs in female and male flowers of monoecious species is completely unknown. A transcriptomic study was performed to investigate the molecular mechanisms behind the defects in the development of the female floral organs of *ga3ox1b*. The results of the RNA sequencing are summarized in Fig. 7 and Supplementary Tables S9–S12. A total of 1036 and 5150 DEGs (|Log_2_FC|>1; adjusted *P*-value <0.05) were found in the corolla and ovary of *ga3ox1b*, respectively, related to those of the WT (Supplementary Table S9). Several biosynthetic GA genes were induced in the mutant corolla and ovary, and two GA-inactivating genes (*GA2ox*) were negatively regulated in the mutant ovary (Fig. 7A; Supplementary Table S10), possibly in an attempt to compensate for GA deficiency. The genes coding for GA receptors GID1B/C and GA negative regulator DELLA proteins GAIPB were up-regulated in the corolla and ovary of *ga3ox1b* compared with the WT.

**Fig. 7.**
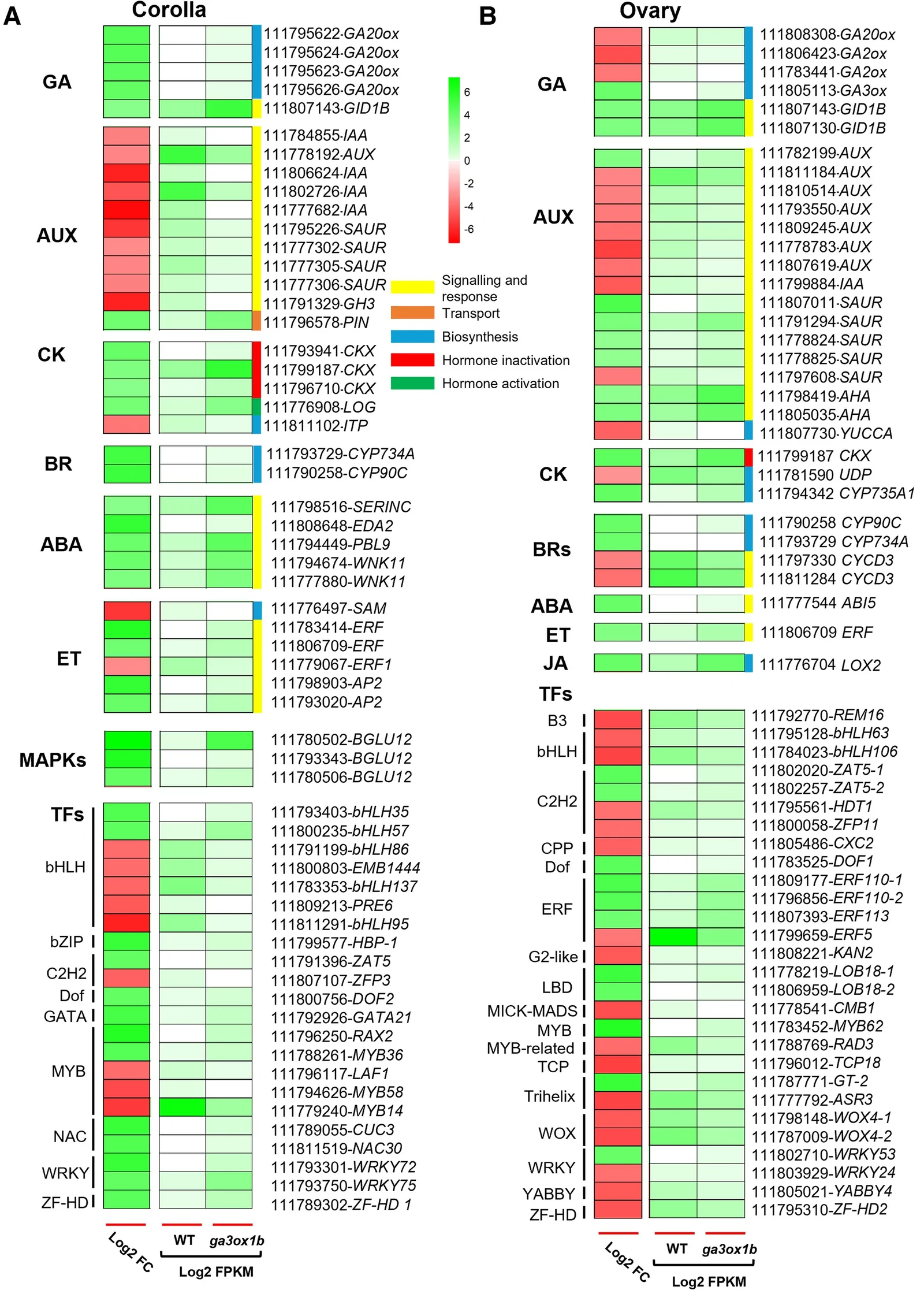
Differentially expressed genes (DEGs) between WT and *ga3ox1b* flowers. (A) Corolla and (B) ovary. Only genes involved in hormonal pathways, MAPK signaling, and transcriptional regulation are shown. Gene functions within each hormonal pathway are indicated by the color key. The heatmaps show log_2_ fold change (log2FC) values, with red representing down-regulated genes and green representing up-regulated genes, and average log2FPKM values for WT and *ga3ox1b* samples. Only genes showing significant differential expression between the WT and *ga3ox1b* in either the corolla or ovary are shown.

In the corolla, it was found that the expression of AUX signaling and response genes including AUX, IAA, SAUR, and GH3-17 was down-regulated in the mutant (Fig. 7A; Supplementary Table S10), indicating that GA positively regulates the AUX signaling pathway during the development of the female corolla. Among the DEGs, no AUX biosynthesis gene was found, validating the similar level of IAA found in the WT and *ga3ox1b* corollas (Fig. 6C). These data suggest that the reduced growth rate of the *ga3ox1b* female petals could be due not only to GA deficiency but also to down-regulation of the AUX signaling pathway. The GA-mediated regulation of cytokinin (CK) and brassinosteroid (BR) in the corolla was not as clear. The slightly higher level of *t*-zeatin in the mutant corolla (Fig. 6E) was correlated with up-regulation of the CK biosynthesis genes, but also with a strong up-regulation of three cytokinin oxidase/dehydrogenases (CKXs) that catalyze oxidative cleavage of CK (Fig. 7A; Supplementary Table S10) to maintain CK homeostasis. Similarly, some BR biosynthesis genes such as CYP734A1 and CYP90C1 were up-regulated in the mutant corolla compared with the WT, but others were down-regulated (Fig. 7A; Supplementary Table S10).

On the other hand, signaling genes for hormones that are negative regulators of plant growth, including ET and ABA, were found to be up-regulated in mutant corolla (Fig. 7A; Supplementary Table S10). The ET biosynthesis gene SAM was down-regulated, but most of the perception and signaling genes were found to be up-regulated in the mutant corolla compared with the WT (Fig. 7A; Supplementary Table S10). The level of ABA in the female corolla was reduced (Fig. 6I), but DEGs of the ABA signaling pathway, including those encoding the serine incorporator, the serine protease EDA2, the serine/threonine protein kinase PBL9, and WNK11, were found to be up-regulated in the *ga3ox1b* corolla (Fig. 7A; Supplementary Table S10). Although the MeJA level was reduced in the mutant corolla (Fig. 6G), only three genes of JA biosynthesis were found to change their expression (Supplementary Table S10).

The transcriptional changes of the phytohormone genes in the ovary of *ga3ox1b* were different from those in the corolla (Fig. 7B; Supplementary Table S10). The AUX biosynthesis gene YUCCA8 was strongly down-regulated, but the AUX signaling pathway was up-regulated, with AUX-negative regulators AUX/IAA repressed, and SAUR-like positive regulator genes activated in the mutant corolla (Fig. 7B; Supplementary Table S10). Most CK biosynthesis and signaling genes, except zeatin *O*-xylosyl transferase, were up-regulated in the mutant ovary (Fig. 7B; Supplementary Table S10). Therefore, it is likely that normal *ga3ox1b* ovarian growth could be caused by the induction of AUX and CK signaling pathways in response to a deficiency in bioactive GA. The role of the BR pathway in ovary growth is not as clear since some DEGs were up-regulated, whereas others were down-regulated (Fig. 7B; Supplementary Table S10).

We have previously reported that ET and JA function as negative regulators of ovary growth in *C. pepo* (García *et al*., 2020; Cebrián *et al*., 2021, 2023). Here, we found that ET biosynthesis, perception, and signaling genes, particularly *ACO1*, *EIN3*, and *ERF1B*, were up-regulated by *ga3ox1b* in the ovary (Fig. 7B; Supplementary Table S10), suggesting that GA negatively regulates ET biosynthesis and signaling in the ovary. Despite this putative induction of the ET signal, the *ga3ox1b* ovary did not reduce its growth (Fig. 4). The biosynthesis and signaling genes of JA and ABA were also found to be up-regulated in the *ga3ox1b* ovary compared with the WT (Fig. 7B; Supplementary Table S10). However, these did not reduce the growth rate of the ovary, which continued to grow, especially in width, in the absence of flower opening and pollination. It is possible that the sensitivity of the ovary to these hormones occurs later in the development of the female flower, probably after petal expansion, flower opening, and pollination.

### Transcription factor genes regulated by *ga3ox1b* in female flowers

Transcription factor (TF) genes that showed significant expression changes between the *ga3ox1b* and WT corollas and ovaries were also identified (Supplementary Table S11). The 54 TFs with log2 FC ≤ −4 or log2 FC ≥ +4 are shown in Fig. 7 and Supplementary Table S12. They were found to be distributed in 18 TF families according to the PlantTF database (Supplementary Table S12). These highly down- and up-regulated TF genes in the GA-deficient mutant should be positive and negative regulators mediating the GA response in the control of female floral organ growth and development.

In the female corolla, positively GA-regulated TF genes included those encoding bHLH137-like (111783353), which has been reported to be responsive to GA and DELLA in Arabidopsis, and bHLH095-like (111811291), which control trichome formation and flower development in tomato by regulating GA biosynthesis genes such as *SlGA20ox2* (Fig. 7A; Supplementary Table S12). Among the negatively GA-regulated TF genes in the corolla are those encoding AP2-like AINTEGUMENTA-like AIL1 (111798903) and AIL5 (111793020), which are involved in the specification of meristematic or division-competent states and in the regulation of the size of floral organs in Arabidopsis, the bZIP protein PERIANTHIA of Arabidopsis or HBP-1b of wheat (111799577), involved in the determination of the number and initiation of floral organs in Arabidopsis, the protein CUP-SHAPED COTYLEDON 3-like (111789055), which may act as an inhibitor of cell division in the control of floral and other organ boundary, and the mini zinc finger protein 1 MIF1 (111789302), which is known as a mediator of plant development by multiple hormones, including GA, ABA, AUX, BR, and CK (Fig. 7A; Supplementary Table S12).

In the ovary, *ga3ox1b*-repressed TF genes can mediate the GA response by acting as positive regulators of ovarian growth and development. These include genes that encode a zinc finger protein 11-like (111800058) and a SUPERMAN-like TF that are involved in the development of various organs, including flowers; the tesmin/TSO1-like protein CXC2, which is expressed in ovules and microspores and is essential for female and male fertility; the ethylene-responsive transcription factor 5-like (111799659), which integrates the signaling pathways of ET and GA into other developmental processes; the TCP18-like TF (111796012), which interacts with DELLA proteins, the major repressors of GA signaling; the trihelix TF ASR3-like (111777792), which is highly induced by GA in rice and involved in plant growth, development, and stress responses; the axial regulator YABBY 4-like (111805021), which mediates parthenocarpic fruit development in cucumber; and the mini zinc finger protein 2-like (111795310) described before (Fig. 7B; Supplementary Table S12).

On the other hand, *ga3ox1b*-induced TF genes could act as negative regulators of GA-mediated ovarian growth and development. These include the genes that encode the following TFs: ABSCISIC ACID-INSENSITIVE 5-like isoform X1 (111777544), an ABA signaling TF that is antagonistic to GA in the control of many plant developmental and stress responses; ethylene-responsive transcription factor (111796856), an ET-responsive TF that also acts as a positive regulator of GA biosynthesis and signaling pathways in Arabidopsis; LOB domain-containing protein 18 (111806959), which is also negatively regulated by GA in Arabidopsis during adventitious root formation; and the TF MYB62-like (111783452), which has been described as a negative regulator of GA biosynthesis and signaling in the regulation of Pi starvation, but also as a TF that can integrate ET and GA pathways in the regulation of female flower determination and floral organ development in *C. pepo* (Fig. 7B and Supplementary Table S12).

### Interaction between ethylene and gibberellin in the regulation of female flower development

Since ET controls the determination of female flowers, we investigated possible interactions between ET and GA in the development of female flowers. The transcriptomic changes between the *ga3ox1b* and WT ovaries were compared with those observed in an RNA-seq study (project No. PRJNA1019239) between the ET-insensitive mutant *etr2b* and the WT ovaries. A total of 3958 DEGs were common to both pairwise comparisons (Supplementary Fig. S4). Among them, we found 89 DEGs for phytohormone biosynthesis and signaling, and 283 DEGs for TFs. Interestingly, all showed completely opposite transcriptional changes in response to *ga3ox1b* or *etr2b* mutations; that is, DEGs induced by ET were repressed by GA, or, conversely, DEGs induced by GA were repressed by ET (Supplementary Tables S13, S14). Thus, we observed a widespread suppression of ABA, ET, GA, JA, and SA pathways, a partial induction of the AUX pathway, and an induction of the BR pathway in *etr2b* ovary, together with the opposite results in *ga3ox1b* ovary (Supplementary Table S13). Several TFs that are highly up-regulated by *ga3ox1b* and down-regulated by *etr2b*, including MYB62, ERF110, or ZAT5, or highly down-regulated by *ga3ox1b* and up-regulated by *etr2b*, including the mini zinc finger protein 2-like and the B3 domain-containing protein At3g19184, are good candidates for integrating the signals of the two hormones during the control of female flower development (Supplementary Table S14). Taken together, these data suggest that GA and ET negatively regulate each other and act antagonistically in the regulation of female flower development in *C. pepo*.

## Discussion

### The gibberellin deficiency of *ga3ox1b* impairs the growth and development of vegetative organs and female flowers

GA3ox catalyzes the conversion of the inactive to the active form of GA (Hedden and Phillips, 2000). This study shows that a 1170 bp DNA insertion in the coding region of *CpGA3ox1B* is the cause of a semi-dwarf phenotype in *C. pepo*. The dwarf phenotype of *ga3ox1b* is characterized by shorter and broader stems, smaller dark green leaves, and shorter petioles, which is consistent with the phenotype reported in other GA-deficient mutants in species such as cucumber (Anarjan *et al*., 2023), rice (Itoh *et al*., 2001), Medicago (Dalmadi *et al*., 2008), maize (Chen *et al*., 2014), and watermelon (Gebremeskel *et al*., 2019; Wei *et al*., 2019). Furthermore, we found that mutant organs have reduced levels of GA_1_ and GA_7_, and that mutant defects could be rescued by external application of GA_3_, concluding that the primary cause of the phenotype is a deficiency in active GA.

In monoecious *C. pepo*, *ga3ox1b* affected the growth and development of the female flower but not that of the male flower. In female flowers, where stamen development is completely arrested at very early stages of development (Martínez and Jamilena, 2021), the GA deficiency reduces petal and pistil growth (not ovary), resulting in female flowers that fail to open and are completely sterile. This contrasts with what has been found in Arabidopsis and other hermaphrodite species, where anther development and male fertility are highly dependent on GA (Nester and Zeevaart, 1988; Jacobsen and Olszewski, 1991; Wilson *et al*., 1992; Cheng *et al*., 2004). The Arabidopsis *ga1-3* mutant shows reduced elongation of pistils, stamens, and petals (Wilson *et al*., 1992). Similarly, flower bud development is delayed in *gib-1*, a gibberellin-deficient tomato mutant in which GA is required for corolla and stamen elongation (Nester and Zeevaart, 1988; Jacobsen and Olszewski, 1991). Reduced expression of the *CpGA3ox1B* gene in male WT corolla (Fig. 2F) would explain the absence of the phenotype in male flowers and indicates that the growth of male floral organs (petals and stamens) is not as dependent on GA as that of female flowers. In fact, during female flower development, the gene is highly expressed in the pistil only at the medium stage of WT flowers (Figs 2F, 4F). These data suggest that local GA biosynthesis in the pistil is required for proper pistil maturation and coordination of petal elongation and flower opening. Supporting this view, studies in cucumber have shown that ovary- or pistil-associated GA biosynthesis is essential for female flower maturation and anthesis, as ovary-derived GA precursors trigger the rapid growth phase leading to flower opening (Pimenta Lange and Lange, 2016).

### *CpGA3ox1B* regulates female flower growth and opening downstream of sex determination mechanisms

The *C. pepo ga3ox1b* mutation did not alter sex determination and sex expression mechanisms and only affected the growth and development of female floral organs, suggesting that GA controls female flower growth after the floral meristem is determined as a female flower. Unisexual flowers of monoecious and dioecious species are known to have evolved from hermaphroditic flowers by acquiring mechanisms that determine the abortion of stamen primordia or carpel primordia into flowers that will eventually develop into female or male flowers, respectively (Martínez and Jamilena, 2021). In *C. pepo*, ET is a feminizing hormone that induces the determination of female flowers by arresting stamen primordia at very early stages of flower development (García *et al*., 2020; Cebrián *et al*., 2021). In contrast, GA has been reported to be a masculinizing hormone that inhibits female flowering and increases the number of male flowers per plant (Segura *et al*., 2023). Although GA is likely to interact antagonistically with ET in determining female flowers, our results suggest that GA biosynthetic enzymes other than *CpGA3ox1B* must have acquired this specific function.

The *ga3ox1b* phenotype clearly indicates that the GA is responsible for the differential growth of male and female flowers. Male flowers, which appear mainly in the basal part of the plant, take ∼30 d to develop and open, while female flowers, which grow mainly in the apical part of the plant, take only ∼14 d (Martínez *et al*., 2013; García *et al*., 2020; Cebrián *et al*., 2021). Therefore, monoecy has led to the co-evolution of differential growth mechanisms between male and female flowers, and this favors the synchronization of flower opening, which facilitates pollination and fertilization. The higher expression of *CpGA3ox1B* and the accumulation of active GA in female flowers (Figs 2F, 6A, B) could enable female flowers in the upper part of the plant to grow faster than male flowers in the lower part.

ET and JA are known to be relevant players in the control of corolla growth and opening in *C. pepo*, although their action is restricted to later stages of flower development. Thus, while *ga3ox1b* shows a reduced corolla growth rate from early stages of female flower development, the insensitive and deficient mutants of ET and JA pathways fail in female petal expansion, a process that occurs 1–2 d before flower opening (García *et al*., 2020; Cebrián *et al*., 2021, 2023; Gautam *et al*., 2024). Therefore, GA positively regulates female corolla growth during early stages of development (Fig. 8). At later stages, ET inhibits GA/AUX/CK signaling and acts upstream of JA, which promotes petal expansion and flower opening (Fig. 8). The latter can render the female flower insensitive to ET and JA in early stages of female flower development. Consequently, no response to external treatments with these two hormones is observed in the *ga3ox1b* mutant or in WT flowers in the earlier and medium stages of development (Cebrián *et al*., 2023). Once the flower has reached the appropriate size, the induction of ET and JA promote petal expansion and flower opening, with ET acting upstream of JA (Cebrián *et al*., 2023; Gautam *et al*., 2024). Strong up-regulation of ET-mediated response TFs such as ethylene-responsive transcription factor 12 (ERF12) and AP2-like ethylene-responsive TFs AINTEGUMENTA-like 1 and 5 (AIL1 and AIL5) in *ga3ox1b* corollas suggests that these factors can integrate ET and GA pathways in the regulation of the development of the female corolla.

**Fig. 8.**
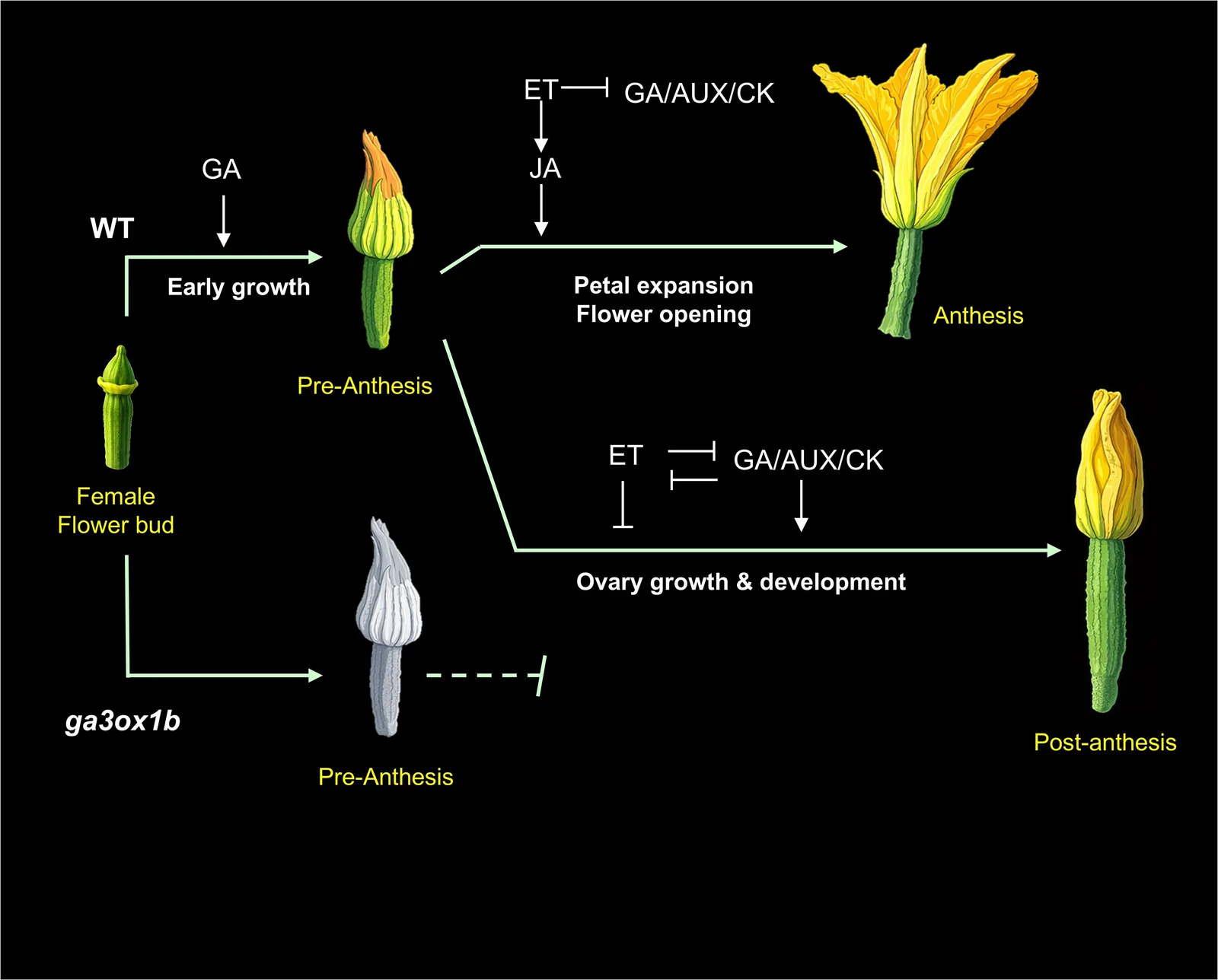
The model illustrates the crosstalk between ethylene (ET)/jasmonic acid (JA) and gibberellins (GAs)/auxins (AUXs)/cytokinins (CKs) during female flower development. During the early stages of growth, GA is required for petal and ovary growth. In *ga3ox1b* plants, flower development is arrested at the pre-anthesis stage and the ovary fails to grow properly. During the later stages of corolla development, ET inhibits GA/AUX/CK signaling and acts upstream of JA, promoting petal expansion and flower opening. Conversely, in the ovary, GA promotes AUX and CK signaling and inhibits ET and JA signaling pathways to maintain growth and development.

### Hormone interactions controlling ovarian development

The growth of the ovary was not coordinated with that of the petals and pistil. As expected for a mutant deficient in GA, the petal and pistil of *ga3ox1b* reduced their growth and never reached the appropriate size, but the ovary of *ga3ox1b* maintained or even increased its growth compared with the WT (Fig. 4). We found no significant changes in hormone levels except for GA in the mutant ovary compared with the WT. However, transcriptomic data showed that GA, AUX, and CK signaling were induced by the *ga3ox1b* mutation in the ovary, which may explain the similar growth of mutant and WT ovaries. ET, JA, and ABA biosynthesis and signaling genes, which are GA antagonists, and act as negative regulators of ovary and fruit development in *C. pepo* (Martínez *et al*., 2013; García *et al*., 2020; Cebrián *et al*., 2023) and other species (de Jong *et al*., 2009; Hou *et al*., 2010), were up-regulated in *ga3ox1b* ovary compared with the WT, although this induction does not appear to be sufficient to inhibit growth in the mutant ovary and fruit.

Furthermore, we have also shown that the ET-insensitive mutation *etr2b* regulates the expression of the same phytohormones and TF genes in a completely opposite manner to *ga3ox1b* (Supplementary Tables S11, S12), indicating that GA and ET negatively regulate each other during the development of the ovary. Therefore, GA promotes AUX and CK signaling but inhibits the ET, JA, and ABA signaling pathways to maintain ovary growth and development. During petal expansion and flower opening, induction of ET and JA inhibits GA biosynthesis and signaling, thereby repressing ovary growth. Therefore, GA induction could mediate enhanced ovary growth of ET- and JA-deficient and insensitive female flowers that delay opening or fail to open (Cebrián *et al*., 2023; García *et al*., 2020; Fig. 8). The *ga3ox1b* ovary can maintain the WT growth rate due to the enhanced signaling of AUX and CK, but also because it did not reach the appropriate size to become sensitive to ET and GA, which coincide with petal expansion and flower opening. In fact, neither ET nor JA was able to rescue the *ga3ox1b* female flower defects.

Comparison of WT, *ga3ox1b*, and *etr2b* transcriptomes led to the identification of a large number of TFs that could mediate crosstalk between GA and ET, among other phytohormones, in the control of ovary development. Several of them are positive regulators of ovarian growth and were down-regulated in *ga3ox1b* but up-regulated in *etr2b*. These include the zinc finger protein 11 (ZFP11), which is involved in cell division and cell elongation. Ectopic expression of the *AtZFP11* gene in tobacco results in dwarfed plants with abnormal leaf morphology, which flower early and are sterile (Dinkins *et al*., 2003). Among the negative regulators of ovarian growth, we found several TFs that were up-regulated in *ga3ox1b* but down-regulated in *etr2b*. These include MYB62 and ERF110 which have been reported to mediate ET-induced female flowering in *C. pepo* (Segura *et al*., 2023, 2024), but also to mediate GA signaling in the regulation of subgynoecy in chieh-qua (Gao *et al*., 2025). Therefore, MYB62 could mediate the ET and GA response during the regulation of ovary growth, but also during the determination of female sex. However, another interesting candidate to integrate both signals is ERF110. In melon and cucumber, ERF110 activates the *ACS11* gene by binding its promoter during the determination of the floral meristem (Tao *et al*., 2018). Furthermore, Arabidopsis ERF110 (also referred to as *AtERF11*) plays an important role in integrating ET and GA pathways, especially in promoting internode elongation by activating GA biosynthesis and signaling genes (Zhou *et al*., 2016).

## Supplementary Material

erag324_Supplementary_Data

## Data Availability

All relevant data can be found within the manuscript and its supplementary data. All of the raw reads generated in this study have been deposited in the public database of National Center of Biotechnology under BioProject PRJNA1427622 (https://dataview.ncbi.nlm.nih.gov/object/PRJNA1427622?reviewer=o24jgo0lsa77bpa55nbqmsisdo).

## Supplementary data

The following supplementary data are available at *JXB* online.

Fig. S1. Molecular characterization of the *CpGA3ox1B* gene.

Fig. S2. Germination and early seedling development.

Fig. S3. Ethylene production of WT and *ga3ox1b* flowers.

Fig. S4. Venn diagram depicting DEGs between *ga3ox1b* and *etr2b* ovaries.

Table S1. Primers used in this study.

Table S2. UPLC-MS/MS conditions for the determination of phytohormones.

Table S3. Inheritance analysis of the *ga3ox1b* mutation.

Table S4. Identified QTLs.

Table S5. Genotyping of an F_2_ population segregating for the *ga3ox1b* phenotype.

Table S6. BLASTp analysis of CpGA3ox1b against *Cucurbita pepo* and *Arabidopsis thaliana* gibberellin oxidases.

Table S7. Evaluation of female and male fertility of *ga3ox1b*.

Table S8. Effect of *ga3ox1b* mutation on sex expression.

Table S9 Total DEGs obtained by RNA-seq.

Table S10 Transcriptomic changes in hormone and MAPK signaling genes.

Table S11. Transcriptomic changes in transcription factor genes.

Table S12. Selected key transcription factor DEGs

Table S13. Common DEGs related to phytohormone pathways in pairwise comparison between the transcriptomes of *ga3ox1b* and WT ovaries and *etr2b* and WT ovaries.

Table S14. Common transcription factor-encoding DEGs obtained by pairwise comparison of the transcriptome of *ga3ox1b* and WT ovaries and that of *etr2b* and WT ovaries.
