# Supplementary material for: The ga3ox1b mutation reveals the crosstalk between gibberellin and other phytohormones in controlling the growth and development of female flowers in Cucurbita pepo
Source: J Exp Bot. 2026 Jul 2;77(19):6522–40. doi: 10.1093/jxb/erag324 (PMC13672989; doi:10.1093/jxb/erag324)
Supplement: Supplementary file 1 [file erag324_supplementary_data.zip › JEXBOT318036-file001.pdf]

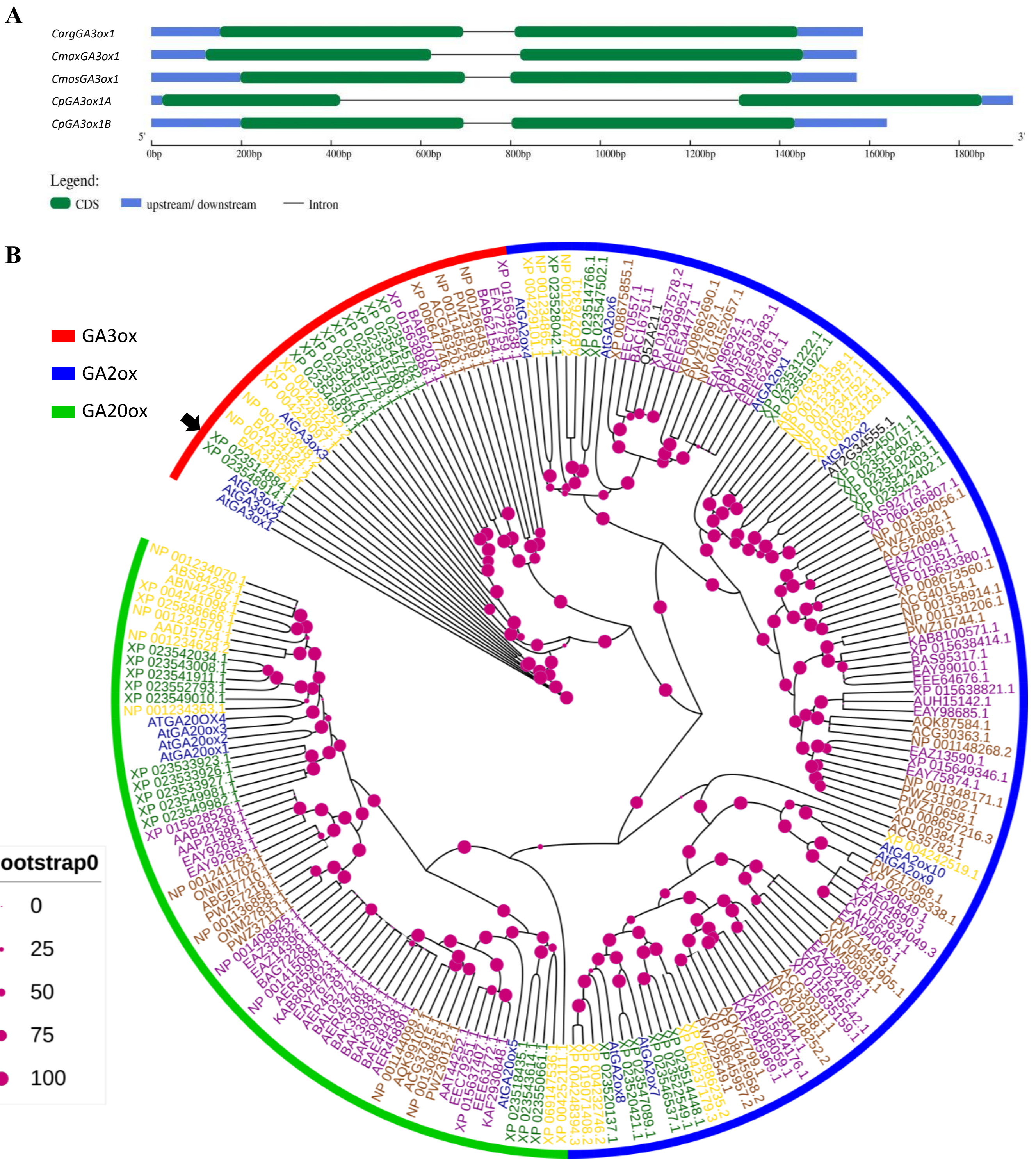

Figure S1. Molecular characterization of the *CpGA3ox1B* gene. **A)** Comparison of the gene structure of *CpGA3ox1B* and its paralog *CpGA3ox1A* with other homologs in *Cucurbita* species. **B)** Phylogenetic tree of GAox proteins identified in *Arabidopsis thaliana* (blue), *Cucurbita pepo* (green), *Solanum lycopersicum* (yellow), *Zea mays* (brown), and *Oryza sativa* (purple). The tree was constructed based on protein sequence alignments and highlights the evolutionary relationships among GAox family members in these species. The black arrow points to the *C. pepo* CpGA3ox1B protein.

A

		WT control	*ga3ox1b* control
% Germination	24 h	100	100
	32 h	100	100
	48 h	100	100
	72 h	100	100

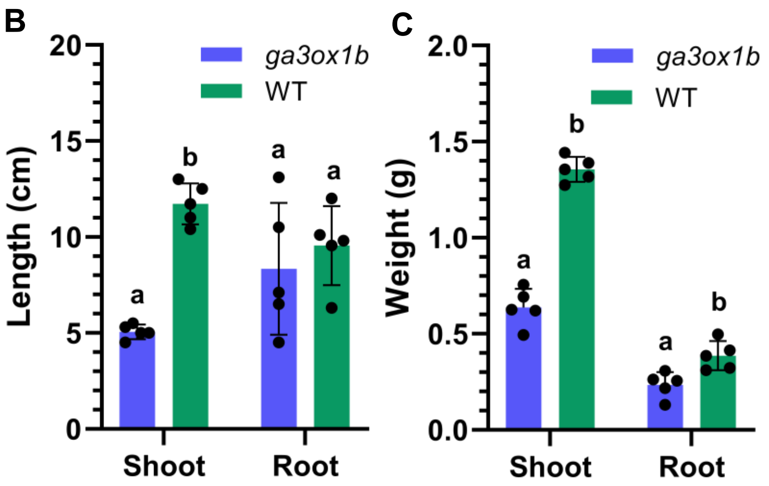

Figure S2. Germination and early seedling development. **A)** Germination rate of WT and mutant seeds. **B)** Shoot and root weight of WT and *ga3ox1b* in darkness for 72 h. Error bars represent SE. Different letters indicate significant differences between genotypes (ANOVA, $P \leq 0.05$).

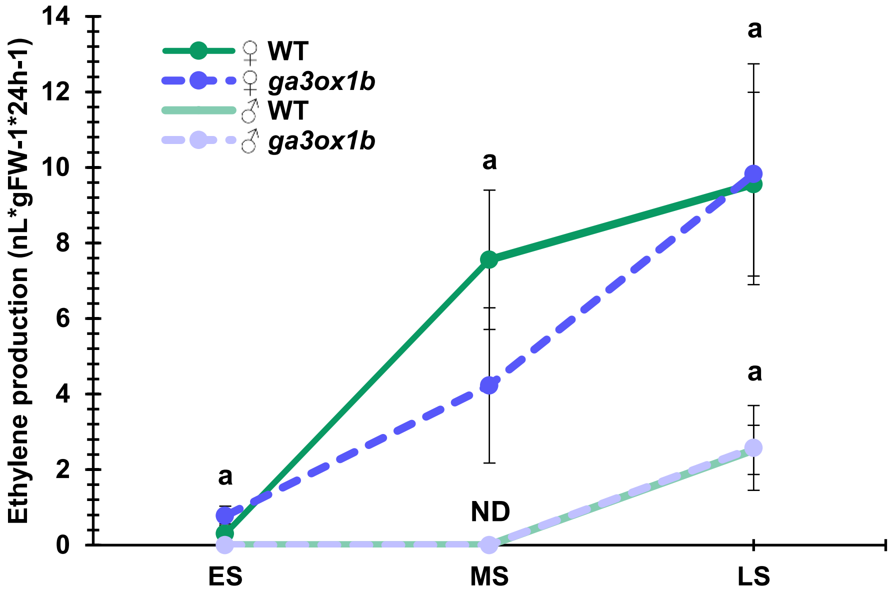

Figure S3. Ethylene production of WT and *ga3ox1b* flowers at ES, MS and LS. Different letters indicate significant differences in ethylene production between genotypes at the same developmental stage (ANOVA, P ≤ 0.05).

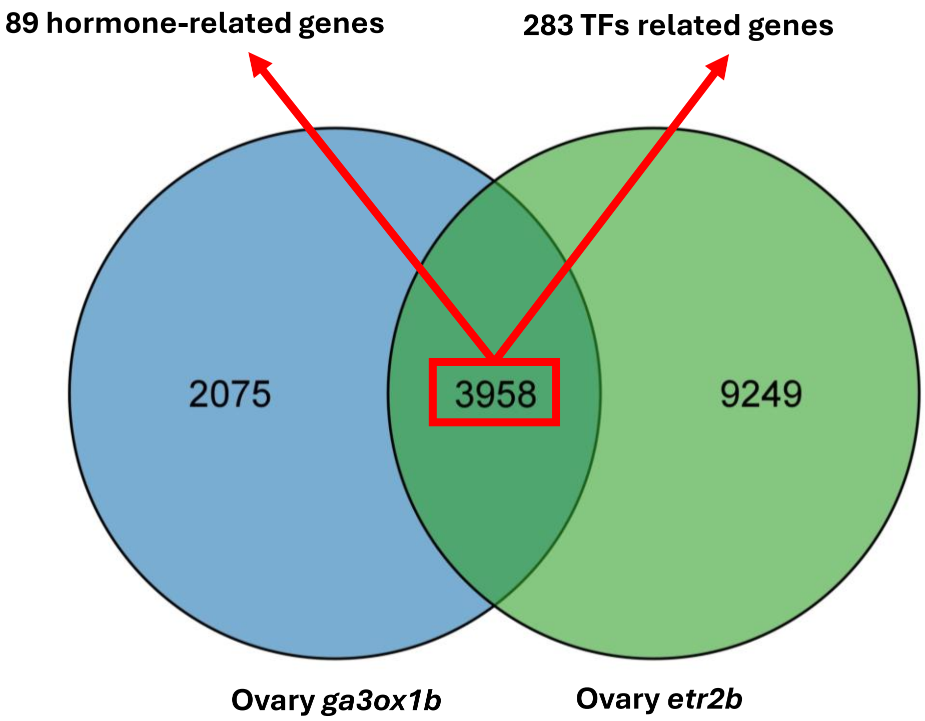

Figure S4. Venn diagram depicting differentially expressed genes (DEGs) between *ga3ox1b* and *etr2b* ovaries.

Table S1. Primers used in this study.

Genotyping		
**Candidate**	**Primer**	**Sequence**
Indel 1	Allele 1-F	GAAGGTGACCAAGTTCATGCTCCGGCCCATTTCATGTCATCC
	Allele 2-F	GAAGGTCGGAGTCAACGGATTCCCGGCCCATTTCATGTCATCA
	Common R	GCGAATTGGGGATAACCCGGGAT
Indel 2	F	GAGCTTCCCGACTCCTATGC
	R	AATAGGAGCGCAAACACGGA
SNP1	Allele 1-F	GAAGGTGACCAAGTTCATGCTTGCTTCTTTGCAACAACGCGG
	Allele 2-F	GAAGGTCGGAGTCAACGGATTCTTGCTTCTTTGCAACAACGCGA
	Common R	GAGCAGCTACATACTCCTTAGACGAA
SNP2	Allele 1-F	GAAGGTGACCAAGTTCATGCTAGTGGTAGGAGTCAAGTTGCTAA
	Allele 2-F	GAAGGTCGGAGTCAACGGATTAGTGGTAGGAGTCAAGTTGCTAC
	Common R	TCAATGGCTCCGGAAGAAACCCTA
**qRT-PCR**		
**Gene**	**Primer**	**Sequence**
CpEF1α	F	CGTCAAGAAGAAATAAGCCA
	R	CTACTACGAGAGAGAGAGCCG
CpACT	F	CCTCTCAATCCCAAAGCTA
	R	CGGCCTGGATAGCAACATA
CpGA3ox1B	F	ACTCCATCAAGCCGTAGTGAA
	R	TCCAGTAATAGGAGCGCAAACA

Table S2. UPLC-MS/MS conditions for the determination of phytohormones.

**Compound**	**Polarity**	**Transition 1 Quantification (m/z)**	**Cone voltage (V)**	**Collision energy (ev)**	**Transition 2 Confirmation (m/z)**	**Cone voltage (V)**	**Collision energy (ev)**
**GA1**	Negative	347.16→259.16	32	18	347.16→273.08	32	24
**GA3**	Negative	345.10→143.04	34	28	345.10→239.14	34	14
**GA4**	Negative	331.16→225.15	32	16	331.16→257.08	32	26
**GA7**	Negative	329.10→223.14	30	18	329.10→267.10	30	10
**Indolacetic acid**	Positive	176.10→130.06	12	14	176.10→103.07	12	28
**Indolbutyric acid**	Positive	204.06→130.09	6	24	204.06→186.07	6	12
**t-zeatin**	Positive	220.08→136.09	24	16	220.08→202.09	24	12
**t-zeatin riboside**	Positive	352.12→220.16	30	16	352.12→136.08	30	34
**Abscisic acid**	Negative	263.10→153.05	14	10	263.10→219.11	14	14
**Methyl jasmonate**	Positive	225.11→133.11	16	12	225.11→151.12	16	12
**Jasmonic acid**	Negative	209.16→58.98	16	12			
**Jasmonate-isoleucine**	Negative	322.11→165.13	32	20	322.11→130.09	32	20
**Salicylic acid**	Negative	136.94→92.99	18	14	136.94→65.03	18	22

**Salicylic acid-d4**	Negative	140.97→97.02	12	16	140.97→69.06	12	24

Table S3. Inheritance analysis of *ga3ox1b* mutation.

**Population**	**Total Plants**	**No. WT plants**	**No. *ga3ox1b* plants**	**Expected segregation**	**χ2**	**P**
P1 (WT)	30	30	0			
P2 (*ga3ox1b*)	30	0	30			
F_1 (P1 x P2)	60	60	0	1:0		
F_2 (F1 x F1)	102	70	32	3:1	0.03	0.85
BC1 (F1 x P2)	34	20	14	1:1	1.05	0.30

Table S7. Evaluation of female and male fertility of *ga3ox1b*. The number of viable seeds per fruit was evaluated in several manual crosses using *ga3ox1b* as the female or male parent.

**♀ Parental (Phenotype)**	**♂ Parental (Phenotype)**	**No. successful crosses (fruits)**	**Average number viable seeds/fruit**
WT	WT	30	292
ga3ox1b	WT	0	0
WT	*ga3ox1b*	8	274
ga3ox1b	*ga3ox1b*	0	0

Table S8. Effect of *ga3ox1b* mutation on sex expression.

**Genotype**	**No. male flowers in 30 nodes**	**Nodes to 1st female flower**
ga3ox1b	9.22 ± 0.99 a	4.77 ± 0.95 a
ga3ox1b/wt	7.85 ± 0.79 a	6.5 ± 0.76 a
WT	9.07 ± 0.77 a	6.2 ± 0.74 a
